# CAFs-derived rho-associated kinase1 mediated EMT to promote laryngeal squamous cell carcinoma metastasis

**DOI:** 10.1186/s12935-023-02911-z

**Published:** 2023-04-16

**Authors:** Liyun Yang, Shiqi Gong, Peipei Qiao, Runyu Zhao, Shuixian Huang, Jieyu Zhou, An Hu

**Affiliations:** 1 Department of Otolaryngology, Gongli Hospital, the Second Military Medical University, Shanghai, 200135 China; 2 Department of Otolaryngology & Head and Neck Surgery, Ruijin Hospital, Shanghai Jiao Tong University School of Medicine, Shanghai, 200025 China; 3 Department of Otolaryngology-Head and Neck Surgery, Shanghai Ninth People’s Hospital, Shanghai Jiaotong University School of Medicine, Shanghai, Ear Institute, Shanghai Jiaotong University School of Medicine, Shanghai, China, Shanghai Key Laboratory of Translational Medicine on Ear and Nose Diseases, Shanghai, 200011 China; 4 Postgraduate Training Base at Shanghai Gongli Hospital, Ningxia Medical University, Shanghai, 200135 China

**Keywords:** CAFs, Metastasis, ROCK1, EMT, JAK2/STAT3/ERK1/2 signal pathway

## Abstract

**Background:**

Cancer-associated fibroblasts (CAFs) play an essential role in tumorigenesis and development of cancers. Nevertheless, the specific molecular mechanism of tumorigenesis and development in Laryngeal squamous cell carcinoma (LSCC) still unknown.

**Methods:**

CAFs, CPFs and NFs were isolated and identified from laryngeal cancer, para-laryngeal cancer and normal tissues. Immunofluorescent staining, Rt-PCR and Western Blot were used to detect the expression of related proteins. Wound healing, migration, invasion and animal experiments were used to examine the ability of movement, migration, invasion and metastasis of LSCC.

**Results:**

ROCK1, was highly expressed in CAFs and CAFs enhanced LSCC metastasis in vivo and vitro, and downregulation of ROCK1 in CAFs inhibited the migration and invasion of LSCC cells. While increasing ROCK1 expression in NFs promoted the migration and invasion of LSCC cells. Further studies revealed that epithelial-mesenchymal transition (EMT) and JAK2/STAT3/ERK1/2 pathway might play an essential role in promoting metastasis of LSCC. In addition, inhibition activity of ROCK1 or JAK2/STAT3/ERK1/2 signal molecules significantly reduced EMT and metastasis.

**Conclusions:**

CAFs-derived ROCK1 via JAK2/STAT3/ERK1/2 axis mediated EMT to promote LSCC metastasis and targeting ROCK1 might provide a potential treatment strategy for LSCC.

**Supplementary Information:**

The online version contains supplementary material available at 10.1186/s12935-023-02911-z.

## Background

Laryngeal squamous cell carcinoma (LSCC) is a type of head and neck cancers [1]. Standard radical resection is now the main treatment for LSCC patients, however, the 5-year survival rate after surgery is not high, mainly because of tumors metastasis [2]. Consequently, it is of great significance to explore the specific molecular mechanism of metastasis for the treatment of LSCC patients.

As a basic process in embryonic development, Epithelial-mesenchymal transition (EMT) is the basis of normal development including wound healing and malignant epithelial neoplasms [3]. EMT is a well characterized embryological process which epithelial cells undergo a phenotypic switch by losing their cell polarity and the epithelial marker (E-catenin, β-catenin) translate into mesenchymal phenotypic cells through acquiring the mesenchymal marker (N-catenin, Vimentin). Losing the polarity of epithelial phenotypes, such as synthesis of basement membranes, can enhance migration and progression and anti-apoptosis [4]. Researches also have found that EMT is involved in tumor metastasis, during the process, tumor cells acquire mesenchymal phenotype and invasiveness through epithelial mesenchymal transformation [5].

Recently, accumulating evidences have manifested interactions between tumor and tumor microenvironment is essential for tumor metastasis. Tumor microenvironment includes tumor and stromal cells [5]. Hence, the interactions between epithelial cells and stromal cells usually act as the regulators of EMT, and the factors creating EMT are originating from the tumor and stromal cells [6]. Normally, tumor stromal cells contain fibroblasts, endothelial, macrophages and lymphocytes cells, which secrete inflammatory factors (TNF-α, TGF-β, IGF), chemokines (IL-8, IL-6, MCP-1), matrix degrading enzyme (MMP-9, MMP-2, MMP-1) and growth factor (EGF) [7]. These factors formed the microenvironment, cancer-associated fibroblasts (CAFs) are the major cells in it which plays the key role in forming the tumor microenvironment and regulating progression and metastasis [8].

Moreover, studies have found that rho-associated kinase1/2 (ROCK1, ROCK2) activation promotes proliferation, however, ROCK1 or ROCK2 inactivation reduces migration [9]. In addition, Stadler S has demonstrated that ROCK1 and ROCK2 paly an essential role in the metastasis of colorectal cancer [10]. Nevertheless, the specific molecular mechanism of tumorigenesis and development in LSCC still unknown.

In our study, we first isolated CAFs from LSCC, and found ROCK1 was highly expressed in CAFs and CAFs enhanced LSCC metastasis in vivo and vitro, and downregulation of ROCK1 in CAFs inhibited migration and invasion of LSCC cells. While increasing ROCK1 expression in normal fibroblasts (NFs) promoted migration and invasion of LSCC cells. Further study indicated that EMT and JAK2/STAT3/ERK1/2 pathway might play an essential role in promoting LSCC metastasis. In addition, inhibition of ROCK1 or JAK2/STAT3/ERK1/2 signal pathway via inhibitors significantly reduced EMT and metastasis. Our study suggested that deprivation of ROCK1 or JAK2/STAT3/ERK1/2 molecules would act as an effective treatment strategy against LSCC.

## Results

### CAFs with high ROCK1 expression enhanced LSCC metastasis

**Fig. 1:**
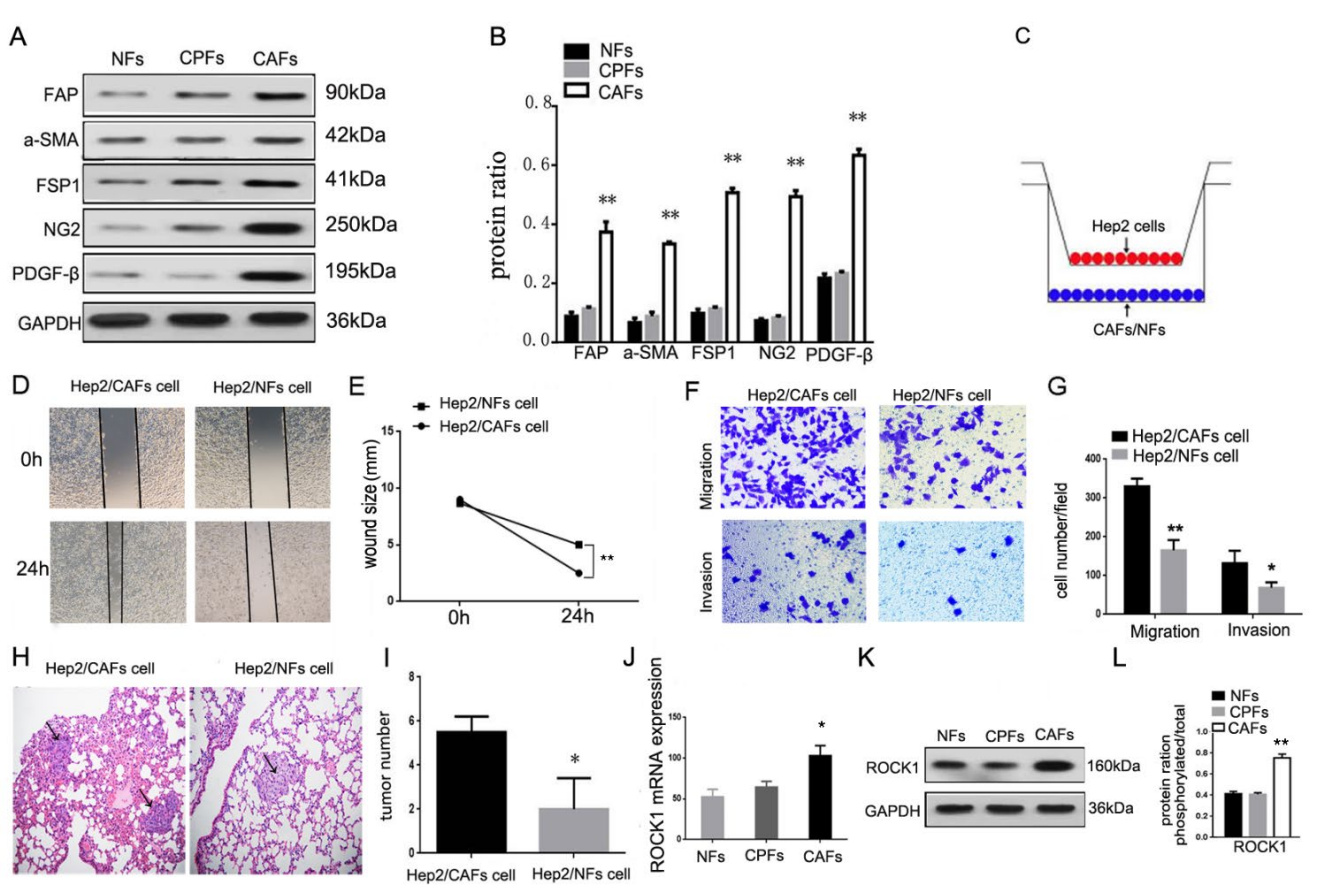
**CAFs were isolated from LSCC and CAFs enhanced the ability of metastasis of LSCC in vitro and vivo and ROCK1 was highly expressed in CAFs. (A)** CAFs specific markers (FAP, α-SMA, FSP1, NG2 and PDGF-β) were determined by Western Blot. The expressions of markers were significantly elevated in CAFs. **(B)** Protein ratio in NFs, CPFs and CAFs (**P < 0.01). **(C)** Co-cultured model was used to separate Hep2 cells line and CAFs or NFs. **(D)** Hep2 cells co-cultured with CAFs or NFs on cell mobility as assessed via wound healing assay. Hep2/CAFs cells increased the mobility. **(E)** Wound size in Hep2/CAFs or NFs cells (**P < 0.01). **(F)** Hep2/CAFs or NFs cells on cell migration and invasion as measured via trans-well assay. Hep2/CAFs cells stimulated the ability of migration and invasion. **(G)** Cells number in every field in Hep2/CAFs or NFs cells (*P < 0.05, **P < 0.01). **(H)** Hep2/CAFs or NFs cells were inoculated into nude mice and pulmonary nodules were observed after six weeks (*N* = 5/group). H&E stains of pulmonary nodules (100×), Hep2/CAFs cells demonstrated larger and more frequently lung metastases as compared to Hep2/NFs cells. **(I)** Pulmonary tissue and nodules were quantified by H&E staining from co-cultured with CAFs or NFs (*P < 0.05). **(J)** The mRNA expressions of ROCK1 were determined by Rt-PCR. (**K).** The protein expression of ROCK1 were determined by Western Blot. （**L).** Protein ratio in NFs, CPFs and CAFs (**P < 0.01)

In our study, CAFs, Cancer Para-laryngeal Fibroblasts (CPFs) and NFs were isolated and identified from laryngeal cancer, para-laryngeal cancer and normal tissues. It is well known that in comparison with NFs, the phenotype of CAFs was significantly different. CAFs express specific molecules, including α-SMA, FSP1, NG2 and PDGF-β receptor, et al. [11, 12]. The specific markers were determined by Western Blot, the expressions of FAP, α-SMA, FSP1, NG2 and PDGF-β were significantly elevated in CAFs (**P < 0.01, Fig. 1A and B). Here, we established a co-culture system in vitro (Fig. 1C), in which fibroblasts were indirectly co-cultured with laryngeal cancer Hep2 cells line, which separated by a semi-permeable membrane (pore size of 0.6 μm). At the appropriate time point, Hep2 cells were either assayed by way of the wound healing, migration, invasion. As showed in Fig. 1D and E, Hep2 co-cultured with CAFs cells (Hep2/CAFs cells) enhanced movement than Hep2 co-cultured with NFs (Hep2/NFs cells, **P < 0.01). Migration assay showed that Hep2/NFs cells (105 ± 8.6) migrated less than Hep2/CAFs cells (286 ± 15.2). Invasion assay also showed similar results, Hep2/NFs cells (51 ± 6.5) invaded less than Hep2/CAFs cells (101 ± 4.0, *P < 0.05, **P < 0.01, Fig. 1F and G). To further illustrate CAFs played a determinant role in metastasis. Hep2/CAFs cells and Hep2/NFs cells respectively inoculated via tail vein into 4-week-old male immunodeficient mice. Six weeks after inoculation, Hep2/CAFs cells (5.8 ± 0.7) demonstrated larger and more frequently lung metastases as compared to Hep2/NFs cells (2 ± 1.5, *P < 0.05, Fig. 1H and I). In our study, the mRNA and protein expressions of ROCK1 considerably high expressed in CAFs in comparison with CPFs and NFs (*P < 0.05, **P < 0.01, Fig. 1J L). Data above indicated that Hep2 cells co-cultured with CAFs compared to Hep2 cells co-cultured with NFs enhanced LSCC metastasis in vivo and vitro and ROCK1 was highly expressed in CAFs.

### ROCK1 enhanced movement, migration and invasion of LSCC cells

**Fig. 2:**
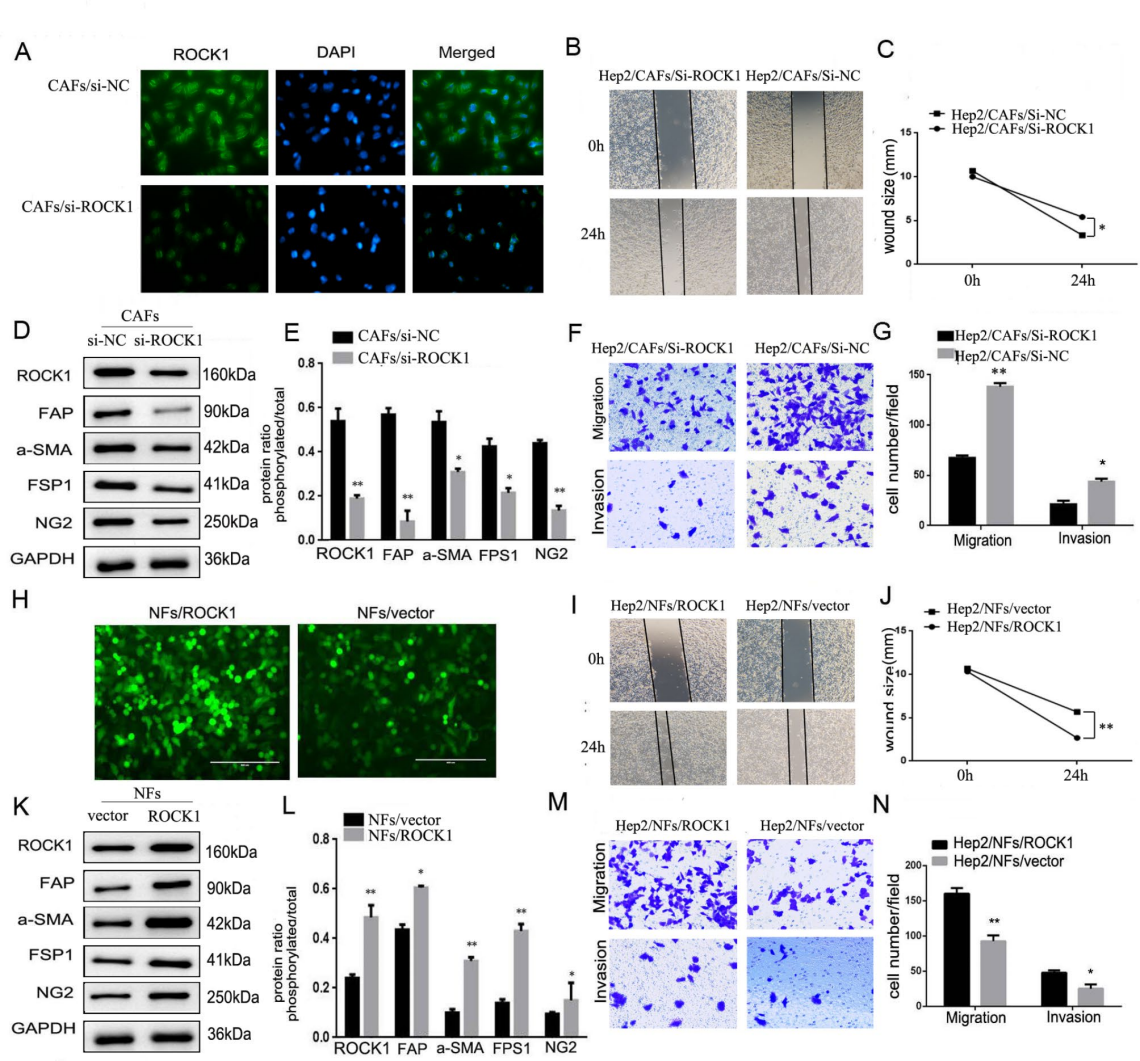
**CAFs enhanced the ability of movement, migration and invasion of Hep2 cells via the high expression of ROCK1. (A)** ROCK1 IF staining in CAFs/si-NC or CAFs/si-ROCK1 cells. ROCK1 level was reduced in Hep2/CAFs/si-ROCK1 cells. (**D).** The inhibition effect and CAFs associative phenotype were verified by Western Blot. ROCK1, FAP, α-SMA, FSP1, NG2 and PDGF-β levels were significantly decreased in Hep2/CAFs/si-ROCK1 cells. (**E).** Protein ratio in CAFs/si-NC and NFs/ROCK1 cells (*P < 0.05, **P < 0.01). **(B)** Hep2/CAFs/si-NC or Hep2/CAFs/si-ROCK1 cells on cell mobility as assessed via wound healing assay. Hep2/CAFs/si-ROCK1 cells decreased the mobility. **(C)** Wound size in Hep2/CAFs/si-NC or Hep2/CAFs/si-ROCK1 cells (*P < 0.05). (**F).** Hep2 cells co-cultured with CAFs/si-NC or CAFs/si-ROCK1 on cell migration and invasion as measured via trans-well assay. Hep2/CAFs/si-ROCK1 inhibit the ability of migration and invasion. (**G).** Cell number in every field in Hep2/NFs/vector and Hep2/CAFs/si-ROCK1 cells (*P < 0.05, **P < 0.01). (**H).** Plasmid transfection was used to up-regulate ROCK1 in NFs, the effect of transfection was showed via IF. (**K).** The expression of ROCK1 and CAFs associative phenotype were verified by Western Blot. ROCK1, FAP, α-SMA, FSP1, NG2 and PDGF-β levels were significantly increased. (**L).** Protein ratio in NFs/vector or NFs/ROCK1 cells (*P < 0.05, **P < 0.01). (**I).** Hep2/NFs/vector or Hep2/NFs/ROCK1 cells on cell mobility as assessed via wound healing assay. Hep2/NFs/ROCK1 cells increased the mobility. (**J).** Wound size in Hep2/CAFs or Hep2/NFs cells (**P < 0.01). (**M).** Hep2/NFs/vector or Hep2/NFs/ROCK1 cells on cell migration and invasion as measured via trans-well assay. Hep2/NFs/ROCK1 cells stimulated the ability of migration and invasion. (**N).** Cell number in every field in Hep2/NFs/vector or Hep2/NFs/ROCK1 cells (*P < 0.05, **P < 0.01)

Considering that ROCK1 was highly expressed in CAFs, and lowly expressed in NFs, it was conceivable that ROCK1 might act as a positive regulator of metastasis. To explore the effect of ROCK1 in contribution on metastasis, ROCK1-siRNA was transfected in CAFs to silence the expression of ROCK1 (CAFs/si-ROCK1), with ROCK1 expression modification confirmed, CAFs were co-cultured with Hep2 cells. Hep2 cells were either assayed by way of the immunofluorescence (IF), Western Blot, wound healing, migration and invasion assays. IF showed that the ROCK1 level was obviously reduced in Hep2 cells co-cultured with CAFs/si-ROCK1 (Hep2/CAFs/si-ROCK1 cells) in comparison with Hep2 cells co-cultured with CAFs/si-NC (Hep2/CAFs/si-NC, Fig. 2A). The results of Western Blot also showed that levels of ROCK1, FAP, α-SMA, FSP1, NG2 and PDGF-β were significantly reduced (*P < 0.05, **P < 0.01, Fig. 2D and E). For wound-healing assay, Hep2/CAFs/si-ROCK1 cells were less motile in comparison with Hep2/CAFs/si-NC cells (*P < 0.05, Fig. 2B C). Similarly, less Hep2/CAFs/si-ROCK1 cells migrated through trans-well chambers (62 ± 8.4) in comparison with Hep2/CAFs/si-NC cells (134 ± 14.5). Ultimately, invasion assay showed that Hep2/CAFs/si-ROCK1 cells (22 ± 7.3) moved across Matrigel less frequently than Hep2/CAFs/si-NC cells (46 ± 6.7, *P < 0.05, **P < 0.01, Fig. 2F and G). All data indicated that deprivation of ROCK1 inhibited movement, migration and invasion of LSCC cells.

Conversely, plasmid transfection was used to increase ROCK1 expression in NFs at the appropriate time point, the effect of transfection was showed in Fig. 2H. Hep2 cells cultured with NFs/ROCK1 (Hep2/NFs/ROCK1 cells) were the experimental group, and Hep2 cells cultured with NFs/vector (Hep2/NFs/vector cells) were the control group. Hep2 cells were either assayed by way of the Western Blot, wound healing, migration and invasion assays. Western Blot results suggested the levels of ROCK1, FAP, α-SMA, FSP1, NG2 and PDGF-β were markedly enhanced (*P < 0.05, **P < 0.01, Fig. 2K L). For wound-healing assay, Hep2/NFs/ROCK1 cells were more motile in comparison with Hep2/NFs/vector cells (**P < 0.01, Fig. 2I J). Similarly, more Hep2/NFs/ROCK1 cells migrated through trans-well chambers (162 ± 8.8) in comparison with Hep2/NFs/vector cells (89 ± 11.4). At last, invasion assay suggested that Hep2/NFs/ROCK1 cells (79 ± 4.24) moved across Matrigel more frequently than Hep2/NFs/vector cells (25 ± 9.7, *P < 0.05, **P < 0.01, Fig. 2M N). All data indicated that deprivation of ROCK1 possessed opposite results. These observations suggested CAFs with high expression of ROCK1 could enhance movement, migration and invasion of LSCC.

### CAFs-derived ROCK1 promoted EMT of LSCC

**Fig. 3:**
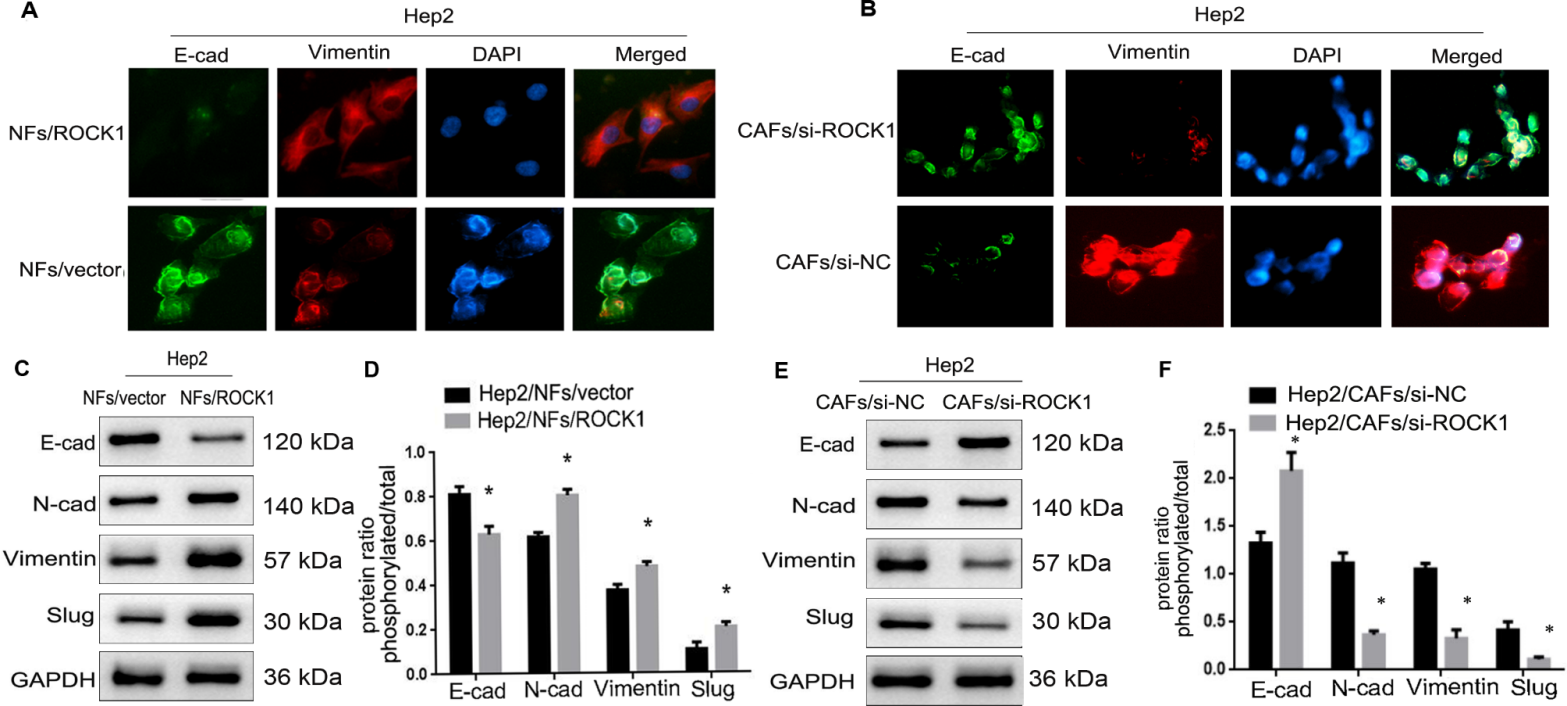
**CAFs-derived ROCK1 promoted the process of EMT of LSCC. (A. B. C. E).** The expressions of E-cadherin and N-cadherin, Slug and Vimentin were detected via IF and Western Blot in Hep2/NFs/ROCK1, Hep2/NFs/vector, Hep2/CAFs/parental and Hep2/CAFs/si-ROCK1 cells. E-cadherin level was reduced in Hep2/NFs/ROCK1 cells, while it was increased in Hep2/CAFs/si-ROCK1 cells. The expressions of N-cadherin, Slug and Vimentin were increased in Hep2/NFs/ROCK1 cells, while they were reduced in Hep2/CAFs/si-ROCK1 cells. (**D.F).** Protein ratio in Hep2/NFs/ROCK1, Hep2/NFs/vector, Hep2/CAFs/parental and Hep2/CAFs/si-ROCK1 cells (*P < 0.05)

Accumulating evidences suggested that EMT, a well-characterized embryological process, had been identified to play a critical role in cancer metastasis [13]. In order to examine the role of CAFs-derived ROCK1 in mediating EMT, Hep2 cells were co-cultured with NFs/ROCK1 (Hep2/NFs/ROCK1 cells), NFs/vector (Hep2/NFs/vector cells), CAFs/si-ROCK1 (Hep2/CAFs/si-ROCK1 cells) or CAFs/parental (Hep2/CAFs/parental cells) in a previously described co-culture system respectively. At the appropriate time point, Hep2 cells were either assayed by way of the IF and Western Blots. As Fig. 3A C and 3D showed that the expression of E-cadherin was markedly decreased, while the expressions of N-cadherin, Slug and Vimentin were observably increased in Hep2/NFs/ROCK1 cells when compared to Hep2/NFs/vector cells (*P < 0.05). In contrast, Fig. 3B and E F showed that expression of E-cadherin was markedly increased, while the expressions of N-cadherin, Slug and Vimentin were markedly decreased in Hep2/CAFs/si-ROCK1 cells when compared to Hep2/CAFs/si-NC cells (*P < 0.05). All data indicated CAFs with high expression of ROCK1 promoted EMT in LSCC.

### Signal molecules of JAK2, STAT3 and ERK1/2 were of great importance in LSCC metastasis

**Fig. 4:**
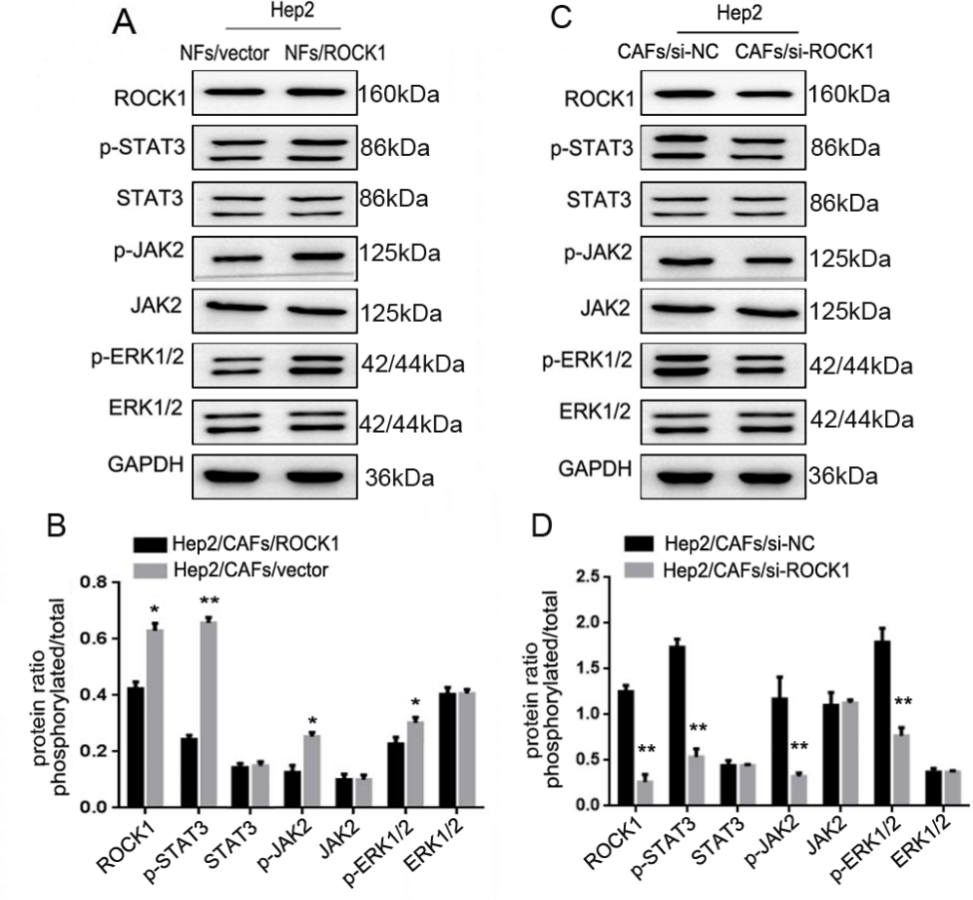
**Signal molecules of JAK2, STAT3 and ERK1/2 were of great importance in the metastasis of LSCC. (A)** The expressions of ROCK1, ROCK2, JAK2, STAT3 and ERK1/2 were verified by Western Blot. The p-JAK2, p-STAT3 and p-ERK1/2 levels were markedly increased in Hep2/NFs/ROCK1 cells. **(B)** Protein ratio in Hep2/NFs/vector or Hep2/NFs/ROCK1 cells (*P < 0.05, **P < 0.01). **(C)** The expressions of ROCK1, ROCK2, JAK2, STAT3 and ERK1/2 were verified by Western Blot in Hep2/CAFs/si-ROCK1 cells. P-JAK2, p-STAT3 and p-ERK1/22 levels were markedly decreased in Hep2/CAFs/si-ROCK1 cells. **(D)** Protein ratio in Hep2/CAFs/si-NC or Hep2/CAFs/si-ROCK1 cells (**P < 0.01)

Tumor metastasis is a multifactorial process [14], signal molecules might play a crucial role in LSCC metastasis. To interrogate the role of signal molecules in CAFs-induced ROCK1 mediating EMT to promote LSCC metastasis. As Fig. 4A and B showed that the expressions of p-JAK2, p-STAT3 and p-ERK1/2 were markedly increased in Hep2/NFs/ROCK1 cells when compared to Hep2/NFs/vector cells (*P < 0.05, **P < 0.01). Consistent with these observations, Fig. 4C and D showed that the expressions of p-JAK2, p-STAT3 and p-ERK1/2 were markedly decreased in Hep2/CAFs/si-ROCK1 cells when compared to Hep2/CAFs/parental cells (**P < 0.01). All data just indicated that signal molecules of JAK2, STAT3 and ERK1/2 were of great importance in LSCC metastasis, while the upstream and downstream relationship between them were not clear.

### CAFs-derived ROCK1 mediated EMT to promote movement, migration and invasion via activating ROCK1/JAK2 axis

**Fig. 5:**
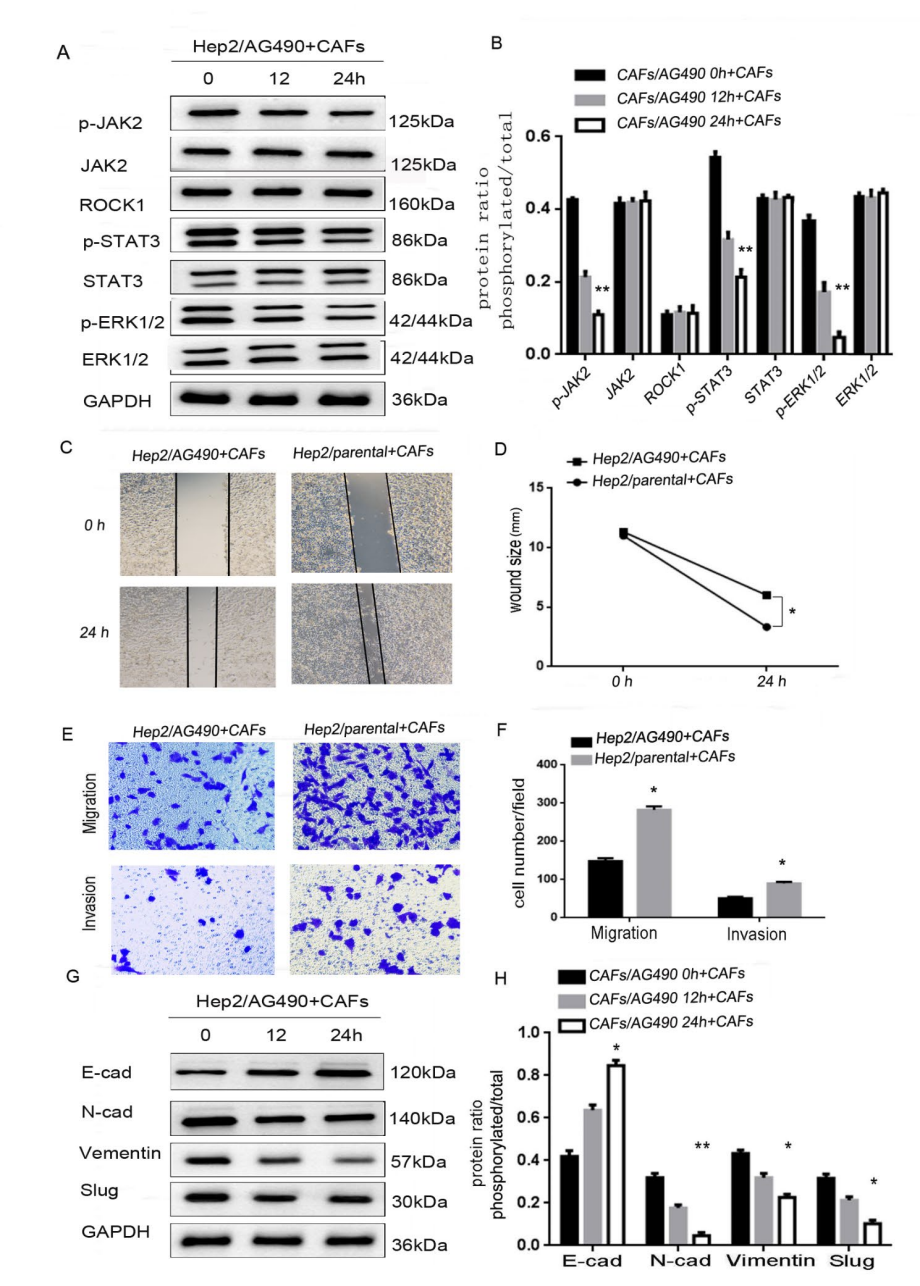
**CAFs-derived ROCK1 mediated the process of EMT to promote the ability of movement, migration, invasion of LSCC via activation of ROCK1/ JAK2 axis. (A)** The expressions of ROCK1, ROCK2, p-JAK2, JAK2, p-STAT3, STAT3, p-ERK1/2 and ERK1/2 were detected by Western Blot in Hep2/AG490 + CAFs cells, levels of p-JAK2, p-STAT3, p-ERK1/2 were markedly decreased, while levels of ROCK1, ROCK2, JAK2, STAT3 and ERK1/2 were not changed. **(B)** Protein ratio in Hep2/AG490 + CAFs cells (**P < 0.01). **(C)** Hep2/parental + CAFs or Hep2/AG490 + CAFs on cell mobility as assessed via wound healing assay. Hep2/AG490 + CAFs cells decreased the mobility. **(D)** Wound size in Hep2/parental + CAFs or Hep2/AG490 + CAFs cells (*P < 0.05). **(E)** Hep2/parental + CAFs or Hep2/AG490 + CAFs cells on cell migration and invasion as measured via trans-well assay. Hep2/AG490 + CAFs cells inhibited the ability of migration and invasion. **(F)** Cell number in every field in Hep2/parental + CAFs or Hep2/AG490 + CAFs cells (*P < 0.05). **(G)** The expressions of E-cadherin and N-cadherin, Slug and Vimentin were detected by Western Blot in Hep2/AG490 + CAFs cells, level of E-cadherin was markedly increased, while levels of N-cadherin, Slug and Vimentin were decreased. **(H)** Protein ratio in Hep2/AG490 + CAFs cells (*P < 0.05, **P < 0.01)

To further verify the upstream and downstream relationship between ROCK1 and signal molecules of JAK2, STAT3 and ERK1/2. One of JAK2 inhibitors, AG490 [15], was used to treat Hep2 cells for 0, 12, 24 h to knockdown the activation of JAK2. With JAK2 expression modification confirmed, the expression of related molecules in Hep2 cells treated with CAFs were examined by Western Blot. As measured by Western Blot, levels of p-JAK2, p-STAT3, p-ERK1/2 were markedly decreased, while levels of ROCK1, ROCK2, JAK2, STAT3 and ERK1/2 were not changed. This indicated that JAK2 was the downstream of ROCK1, and the upstream of STAT3 and ERK1/2 (**P < 0.01, Fig. 5A and B). We further examined whether blocking JAK2 by AG490 could also inhibit the tumor-promoting effects on LSCC. In wound-healing assay, Hep2/AG490 + CAFs cells were less motile at 24 h in comparison with Hep2/parental + CAFs (*P < 0.05, Fig. 5C and D). Similarly, less Hep2/AG490 + CAFs cells migrated through trans-well chambers (67 ± 6.8) in comparison with Hep2/parental + CAFs cells (143 ± 7.2). In the end, invasion assay showed Hep2/AG490 + CAFs cells (32 ± 10.5) moved less frequently than Hep2/parental + CAFs cells (63 ± 12.7, *P < 0.05, Fig. 5E F). These observations matched the significant increase in E-cadherin expression, while the expressions of N-cadherin, Slug and Vimentin were significantly decreased (Fig. 5G H, *P < 0.05, **P < 0.01). All data illustrated that CAFs-derived ROCK1 mediated EMT to reinforce movement, migration and invasion via activating ROCK1/JAK2 axis.

### CAFs-derived ROCK1 mediated EMT to promote movement, migration and invasion via activating ROCK1/JAK2/ STAT3 axis

**Fig. 6:**
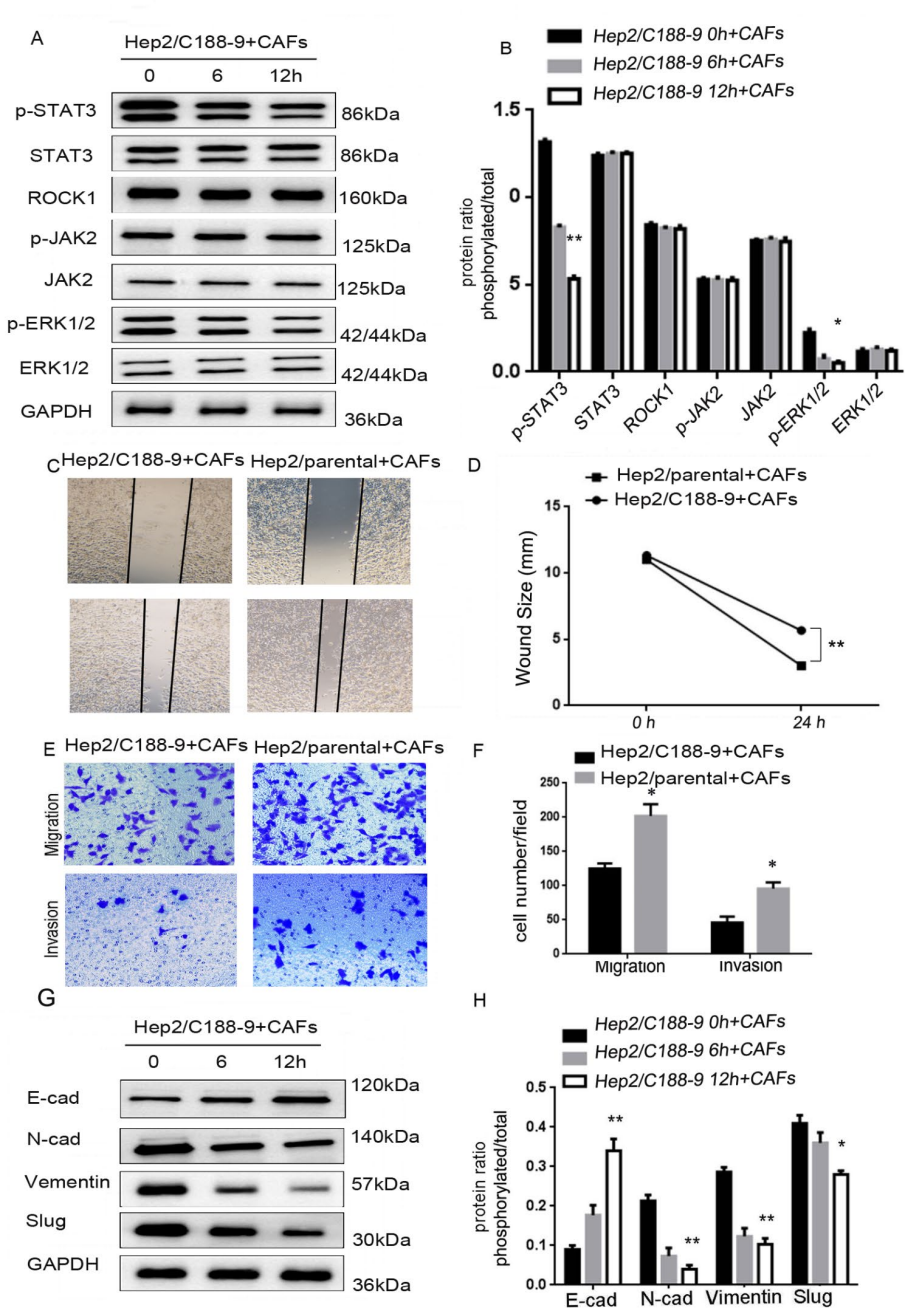
**CAFs-derived ROCK1 mediated the process of EMT and enhanced the ability of movement, migration, invasion of LSCC via the activation of ROCK1/JAK2/STAT3 signal pathway. (A)** The expressions of ROCK1, ROCK2, p**-**JAK2, JAK2, p-STAT3, STAT3, p-ERK1/2 and ERK1/2 were detected by Western Blot in Hep2/C188-9 + CAFs cells, levels of p-STAT3, p-ERK1/2 were markedly decreased, while levels of ROCK1, ROCK2, p-JAK2, JAK2, STAT3 and ERK1/2 were not changed. **(B)** Protein ratio in Hep2/C188-9 + CAFs cells (*P < 0.05, **P < 0.01). **(C)** Hep2/parental + CAFs or Hep2/C188-9 + CAFs cells on cell mobility as assessed via wound healing assay. Hep2/C188-9 + CAFs cells decreased the mobility. **(D)** Wound size in Hep2/parental + CAFs or Hep2/C188-9 + CAFs cells (**P < 0.01). **(E)** Hep2/CAFs/ parental or Hep2/C188-9 + CAFs cells on cell migration and invasion as measured via trans-well assay. Hep2/C188-9 + CAFs cells inhibited the ability of migration and invasion. **(F)** Cell number in every field in Hep2/parental + CAFs or Hep2/C188-9 + CAFs cells (*P < 0.05). **(G)** The expressions of E-cadherin and N-cadherin, Slug and Vimentin were detected via Western Blot in Hep2/C188-9 + CAFs cells, level of E-cadherin was markedly increased, while levels of N-cadherin, Slug and Vimentin were decreased. **(H)** Protein ratio in Hep2/C188-9 + CAFs cells (*P < 0.05, **P < 0.01)

To elucidate the role of STAT3 in LSCC metastasis. In the same way, one STAT3 inhibitor, C188-9 [16], was used to treat Hep2 cells for 0, 6, 12 h to knockdown the activation of STAT3. With STAT3 expression modification confirmed, the expression of related molecules in Hep2 cells treated with CAFs were examined by Western Blot. Western Blot showed that the levels of p-STAT3, p-ERK1/2 were markedly decreased, while levels of ROCK1, ROCK2, p-JAK2, JAK2, p-STAT3, STAT3, p-ERK1/2and ERK1/2 were not changed (*P < 0.05, **P < 0.01, Fig. 6A and B). This indicated that STAT3 was the downstream of ROCK1/JAK2, and was the upstream of ERK1/2. We further examined whether blocking STAT3 expression could restrain LSCC metastasis as well. In wound-healing assay, Hep2/C188-9 + CAFs cells were less motile at 24 h in comparison with Hep2/parental + CAFs (**P < 0.01, Fig. 6C and D). Similarly, less Hep2/C188-9 + CAFs cells migrated through trans-well chambers (46 ± 6.4) in comparison with Hep2/parental + CAFs cells (92 ± 6.8). Finally, invasion assay indicated that Hep2/C188-9 + CAFs cells (29 ± 6.1) moved through Matrigel less frequently than Hep2/parental + CAFs cells (44 ± 4.3, *P < 0.05, Fig. 6E F). Consistent with these observations, Fig. 6G H showed that the level of E-cadherin was markedly increased, nevertheless the levels of N-cadherin, Slug and Vimentin were markedly decreased (*P < 0.05, **P < 0.01). Similarly, these data indicated CAFs-derived ROCK1 mediated EMT to promote movement, migration, invasion via activating ROCK1/JAK2/STAT3 signal pathway.

### CAFs-derived ROCK1 mediated EMT to promote movement, migration and invasion via activating ROCK1/JAK2/STAT3/ERK1/2 axis

**Fig. 7:**
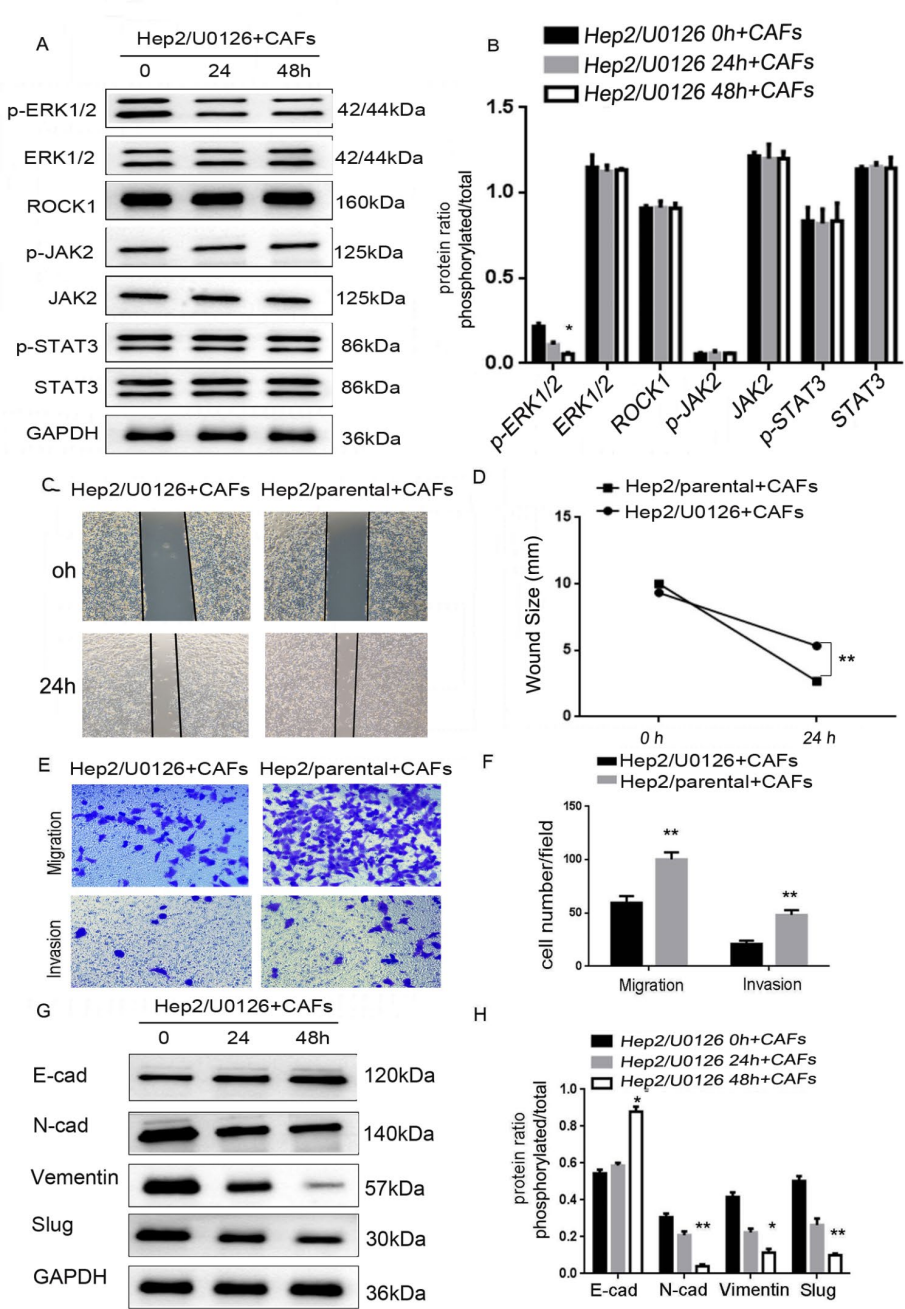
**CAFs-derived ROCK1 mediated the process of EMT and promoted the ability of movement, migration, invasion of LSCC via the activation of ROCK1/JAK2/STAT3/ERK1/2 axis. (A)** The expressions of ROCK1, ROCK2, p-JAK2, JAK2, p-STAT3, STAT3, p-ERK1/2 and ERK1/2 were detected by Western Blot in Hep2/U0126 + CAFs cells, levels of p-ERK1/2 were markedly decreased, while levels of ROCK1, ROCK2, JAK2, p-JAK2, p-STAT3, STAT3 and ERK1/2 were not changed. **(B)** Protein ratio in Hep2/U0126 + CAFs cells (*P < 0.05). **(C)** Hep2/parental + CAFs or Hep2/U0126 + CAFs cells on cell mobility as assessed via wound healing assay. Hep2/U0126 + CAFs cells decreased the mobility. **(D)** Wound size in Hep2/parental + CAFs or Hep2/U0126 + CAFs cells (**P < 0.01). **(E)** Hep2/parental + CAFs or Hep2/U0126 + CAFs cells on cell migration and invasion as measured via trans-well assay. Hep2/U0126 + CAFs cells inhibited the ability of migration and invasion. **(F)** Cell number in every field in Hep2/parental + CAFs or Hep2/U0126 + CAFs cells (**P < 0.01). **(G)** The expressions of E-cadherin and N-cadherin, Slug and Vimentin were detected via Western Blot in Hep2/U0126 + CAFs cells, level of E-cadherin was markedly increased, while levels of N-cadherin, Slug and Vimentin were decreased. **(H)** Protein ratio in Hep2/U0126 + CAFs cells (*P < 0.05, **P < 0.01)

Similarly, one ERK1/2 inhibitor, U0126 [17], was used to treat Hep2 cells for 0, 24, 48 h to block activation of ERK1/2. With ERK1/2 expression modification confirmed, the expression of related molecules in Hep2 cells treated with CAFs were examined by Western Blot. Western Blot showed that the level of p-ERK1/2 was markedly decreased, while the levels of ROCK1, ROCK2, p-JAK2, JAK2, p-STAT3, STAT3, and ERK1/2 were not changed (*P < 0.05, Fig. 7A and B). This indicated that ERK1/2 was the downstream of ROCK1/JAK2/STAT3. We further examined whether blocking ERK1/2 expression could also inhibit the tumor-promoting effects on LSCC. In wound-healing assay, Hep2/U0126 + CAFs cells were less motile at 24 h in comparison with Hep2/parental + CAFs (**P < 0.01, Fig. 7C and D). Similarly, less Hep2/U0126 + CAFs cells migrated through trans-well chambers (67 ± 10.3) in comparison with Hep2/parental + CAFs cells (91 ± 11.7). Ultimately, the results of invasion assay manifested that Hep2/U0126 + CAFs cells (23 ± 4.8) moved through Matrigel less frequently than Hep2/parental + CAFs cells (49 ± 6.5, **P < 0.01, Fig. 7E F). These observations match what the expression of E-cadherin was markedly increased, while the expressions of N-cadherin, Slug and Vimentin were markedly decreased (*P < 0.05, **P < 0.01, Fig. 7G H). These data indicated CAFs-derived ROCK1 mediated EMT to aggrandize the movement, migration and invasion ability of LSCC via activating ROCK1/JAK2/STAT3/ERK1/2 axis.

### Blocking ROCK1 axis impaired LSCC metastasis induced by CAFs *in vivo*

**Fig. 8:**
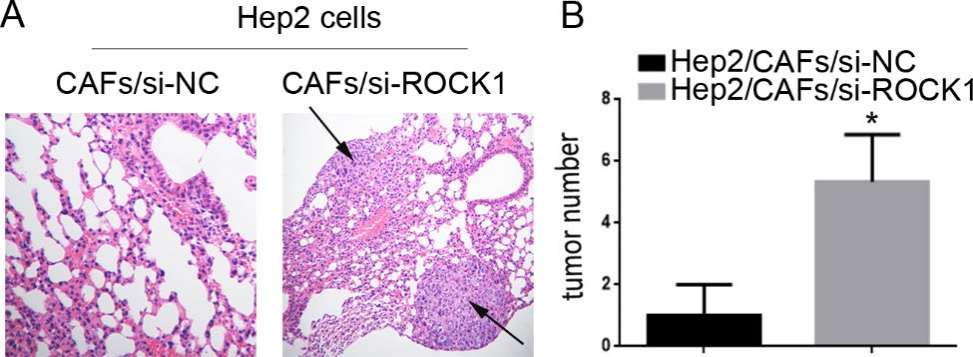
**Blocking ROCK1 induced by CAFs impaired LSCC metastasis in vivo. (A)** Hep2 cells, co-cultured with CAFs/si-ROCK1, were inoculated into nude mice and pulmonary nodules were observed after four weeks (N = 5/group), Hep2 cells co-cultured with CAFs/si-NC were control group. H&E stains of pulmonary nodules (100×), Hep2/CAFs/si-ROCK1 cells demonstrated less frequently lung metastases as compared to Hep2/CAFs/si-NC cells. **(B)** Pulmonary tissue and nodules were quantified by H&E staining (*P < 0.05)

In our study, we further estimated the contribution of ROCK1 on LSCC metastasis in vivo. Hep2 cells, co-cultured with CAFs/si-ROCK1 were experimental groups, Hep2 cells co-cultured with CAFs/si-NC were control groups. As showed in Fig. 8A and B, Hep2/CAFs/si-NC (5 ± 1.7) demonstrated more frequently lung metastases compared with Hep2/CAFs/si-ROCK1 (1 ± 1, *P < 0.05). All results suggested that CAFs-derived ROCK1 promoted LSCC metastasis in vivo.

## Discussion

Interactions between tumor and tumor microenvironment is essential for tumor metastasis. Microenvironment includes tumor and stromal cells [5]. CAFs, the activated fibroblasts in tumor stroma, is important modifiers of tumor progression. CAFs are known to be a heterogeneous group of cells, mainly derives from normal epithelial cells, mesenchymal fibroblasts, bone marrow mesenchymal stem cells, vascular beds, or epithelium. In comparison with NFs, the phenotype and function were significantly different. CAFs can express specific molecules, α-SMA, FAP, FSP1/S100A4, NG2 and PDGF-receptor, et al. [19]. CAFs, which act as a “stromal response”, plays a prominent role in tumor microenvironment [20]. Therefore, a better understanding with the molecule mechanism of metastasis is of great significance for developing effective clinical prevention programs and new targeted therapies for LSCC.

First and foremost, we isolated CAFs, CPFs and NFs from laryngeal cancer tissues, para-laryngeal cancer tissues and normal tissues. The specific markers were determined by Western Blot, CAFs highly expressed of α-SMA, FAP, NG2 and FSP1 and enhanced LSCC metastasis in vitro and vivo. Interestingly, we found ROCK1 was highly expressed in CAFs. This might indicate that CAFs with the high expression of ROCK1 played a core effect in enhancing movement, migration and invasion of LSCC.

It was well known that ROCK1/2 played a role in regulating cell cycle, proliferation and mitosis. Activation of ROCK1 could induce cell proliferation, inversely, inhibition ROCK1 could reduce migration [9]. Stadler et al. also found that CAFs could promote colorectal cancer metastasis through ROCK1 [10]. Here, we co-cultured CAFs and NFs with laryngeal cancer Hep2 cell line respectively. We found ROCK1 was highly expressed in CAFs, and ROCK1 might promote LSCC metastasis in vivo and vitro. To further explore CAFs-derived ROCK1 promoted LSCC metastasis, we down-regulated ROCK1 expression in CAFs and up-regulated ROCK1 expression in NFs respectively, the results showed that the expressions of CAFs associative phenotypes markedly decreased, and the ability of movement, migration and invasion was also obviously reduced in Hep2 cells co-cultured with deprivation of ROCK1 in CAFs. On the contrary, the expressions of CAFs associative phenotypes markedly increased, and the ability of movement, migration and invasion was also obviously increased in Hep2/NFs/ROCK1 cells. All that indicated that CAFs-derived ROCK1 authentically promoted LSCC metastasis.

Accumulating evidence have indicated EMT, characterized by loss of polarity of epithelial cells, is important modifiers of tumor progression. The epithelial markers (E-cadherin, β-catenin) acquired the mesenchymal markers (N-cadherin, vimentin, ZEB2) to form mesenchymal cells thus these transformed epithelial cells acquire fibroblast like properties and exhibit reduced cell-cell adhesion and increased motility [21]. In order to examine the role of CAF-derived ROCK1 in contemplating EMT in LSCC, IF and Western Blot were applied to inspect the expressions of EMT associated phenotype markers in Hep2/NFs/ROCK1, Hep2/NFs/vector, Hep2/CAFs/parental and Hep2/CAFs/si-ROCK1 cells respectively. Results showed that epithelial phenotype marker (E-cadherin) levels were markedly decreased, and mesenchymal phenotype markers (N-cadherin, Slug and Vimentin) levels were markedly increased in Hep2/NFs/ROCK1 cells. The results were reversed in Hep2/CAFs/si-ROCK1 cells, which indicated that CAFs-derived ROCK1 mediated EMT to promote LSCC metastasis.

Furthermore, to further explore the molecular mechanism of ROCK1 mediated EMT to accelerate LSCC metastasis. Signal molecules play a crucial role in LSCC metastasis. Signal molecular pathway of JAK2 played an important role in cancer metastasis of cancer. The expression of STAT3 and phosphorylated STAT3 increased in gastric cancer in comparison with normal stomach [22]. The activation of STAT3 was positive in early gastric cancer, poorly differentiated adenocarcinoma and metastatic lymph node tissues [24]. ERK1/2 was generally located in cytoplasm, but when it was activated, it could across the nuclear membrane and transfer to nucleus [24]. ERK1/2 could induce cyclin D1 expression and accelerated cell mitosis to promote cell proliferation [26].

To further interrogate the role of signal molecules in mediating CAFs-induced ROCK1 activated EMT to promote LSCC metastasis. Here, we first explored the activation of JAK2, STAT3 and ERK1/2 in Hep2/NF/ROCK1 and Hep2/CAFs/si-ROCK1 cells respectively. Results of Western Blot showed that p-JAK2, p-STAT3 and p-ERK1/2 levels were markedly decreased in Hep2/CAFs/si-ROCK1 cells. Inversely, p-JAK2, p-STAT3 and p-ERK1/2 levels were markedly increased in Hep2/NFs/ROCK1 cells. All data indicated that signal molecules of JAK2, STAT3 and ERK1/2 were of great importance in LSCC metastasis, while the upstream and downstream relationships were not clear. In order to explore above problem, inhibitors of JAK2, STAT3 and ERK1/2 (AG490, C188-9, U0126) were applied. All data showed that JAK2 was the downstream of ROCK1, and the upstream of STAT3 and ERK1/2. STAT3 was the downstream of ROCK1/JAK2, and the upstream of ERK1/2. ERK1/2 was the downstream of ROCK1/JAK2/ERK1/2. Ultimately, blocking ROCK1/JAK2/STAT3/ERK1/2 pathway impaired the metastasis of LSCC cells.

In summary, ROCK1 was highly expressed in laryngeal CAFs, CAFs-derived ROCK1 mediated EMT via activating JAK2/STAT3/ERK1/2 signal pathway to promote LSCC metastasis. These findings exhibited that stromal ROCK1 could enhance LSCC initiation and progression, and targeting ROCK1 might provide a potential treatment stratagem for LSCC.

## Materials and methods

### Cell cultures

Human laryngeal cancer Hep2 cell lines were preserved in our laboratory. Cells were repeatedly cultured in DMEM with 10% FBS containing 100 IU/ml penicillin and 100 IU/ml streptomycin in a humidified cell incubator with an atmosphere of 5% CO_2_ at 37 °C. Laryngeal cancer tissue was cut from the edge of the tumor, para-cancer tissue was taken from 4 to 5 cm away from the edge of the tumor and adjacent non-tumor tissues was derived from the normal tissue of the larynx. Tumor tissues, adjacent tissues and adjacent non-tumor tissues were mechanically minced into small pieces (1–1.5 mm^3^) and seeded onto 10 cm petri dishes in DMEM with 10% FBS containing 100 IU/ml penicillin and 100 IU/ml streptomycin. A homogeneous group of fibroblasts, produced after 7–14 days of culture, would be used in the experiments.

### Western blot

Cells were lysed with RIPA buffer (Thermo Fischer Scientific). The BCA Protein Assay Kit (Pierce, Rockford, USA) was used to measure protein concentrations. 10% SDSPAGE was used to separate 100 µg proteins for 2 h and the proteins were transferred onto PVDF membranes (Millipore, MA, USA). Blocking membranes with 5% nonfat milk in 1×TBST for 2 h and incubating with primary antibodies overnight at 4 °C. Antibodies included anti-FAP, anti-SAM, anti-FSP1, anti-NG2, anti-PDGF-β, anti-ROCK1, anti-ROCK2, anti-E-cad, anti-N-cad, anti-Vimentin, anti-Slug, anti-p-JAK2, anti-JAK2, anti-p-STAT3, anti-STAT3, anti-p-ERK1/2, anti-ERK1/2, GAPDH and Secondary antibody which were purchased from Abcam, Cambridge, MA, USA. A Tanon 5200 system was used to visualize proteins.

### Immunofluorescent staining

5 × 10^4^ cells were seeded into slides (Millipore, MA, USA) and fixed with 4% paraformaldehyde (PFA) for 30 min. Slides were rinsed with PBS for 3 times, blocking slides with 5% BSA for 1 h at room temperature and incubating slides with primary antibodies at 4 °C overnight. Next day, rinsing slides with PBS for 3 times and incubating slides with secondary antibodies in the dark at room temperature for 1 h. Antibodies included anti-ROCK1, anti-E-cad and anti-Vimentin, Alexa Fluor® 488 goat and Alexa Fluor® 555 which were purchased from Abcam, Cambridge, MA, USA were used to IF staining. Visualizing nuclei with DAPI in the dark for 5 min. Analyzing slides by fluorescent microscopy (10x).

### Transfections

Seeding 3 × 10^5^ cells into 6-well plates and incubating cells overnight. Transfecting cells with ROCK1 plasmid (Myc-ROCK1-Delta3 (1-727), Gene Pharma Company, Shanghai) by lipofectamine 2000 (Invitrogen) and selecting 1200 ug/ml G418. Verifying selected clones with Western Blot and frozen.

### Wound healing assay

Inoculating 1 × 10^6^ cells into 6-well plates and scratching cells by using 20 µl pipette tips after overnight incubation. Then, photographing cells by using a high-powered microscope (2x).

### Cell migration and invasion assay

Seeding 2 × 10^5^ Hep2 cells seeded into the upper chambers of trans-wells (Boyden trans-well chambers, Corning, MA, USA) with 200 ul of serum-free DMEM and adding 3 × 10^4^ CAFs with or without inhibitors in 800ul medium containing 10% fetal calf serum into the lower chambers. Culturing cells for one day and staining filters with crystal violet at room temperature for 30 min. For invasion assay, coating upper chamber membranes in Matrigel (Becton Dickinson Labware, Bedford, MA, USA). The membranes of above layer were stained with crystal violet. Counting cells by using a high-power objective (10 x) in five random fields.

### In vivo metastasis

Hep2 cells, with the different treatments, in 200 µl PBS were injected into the tail vein of these 4-week-old male nude mice (the Institute of Zoology, Chinese Academy of Sciences). Mice was randomly assigned to 2 groups (five in each group). After six weeks, excess pentobarbital sodium (4%, 200 mg/kg; Sigma, Shanghai, China) were used to injected into these nude mice. The lungs were collected for H&E. The numbers of lung metastases were calculated at the magnification (×100).

### Statistical analysis

Analyze data by Graph-Pad Prism 6 software and displaying by means ± SD. One-way analysis of variance (ANOVA) was used to analyze the data and the significance level was set at P < 0.05.

## Electronic supplementary material

Below is the link to the electronic supplementary material.

Supplementary Material 1

## Data Availability

The data and material during the current study were available from the corresponding author on reasonable request.

## Abbreviations

LSCC: Laryngeal squamous cell carcinoma
HNSCC: Head and neck squamous cell carcinoma
CAFs: Cancer-associated fibroblasts
CPFs: Cancer Para-laryngeal Fibroblasts
NFs: Normal Fibroblasts
EMT: Epithelial-mesenchymal transition
ROCK1: Rho-associated kinase1
IF: immunofluorescence
