# Supplementary figures and images for: CAFs-derived rho-associated kinase1 mediated EMT to promote laryngeal squamous cell carcinoma metastasis
Source: Cancer Cell Int. 2023 Apr 16;23:70. doi: 10.1186/s12935-023-02911-z (PMC10105957; doi:10.1186/s12935-023-02911-z)

Figure1

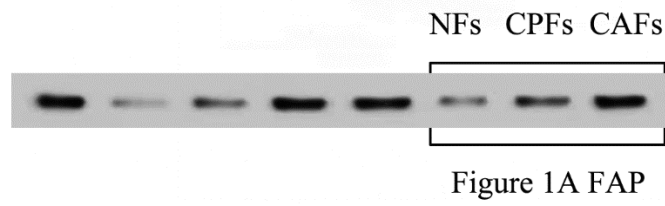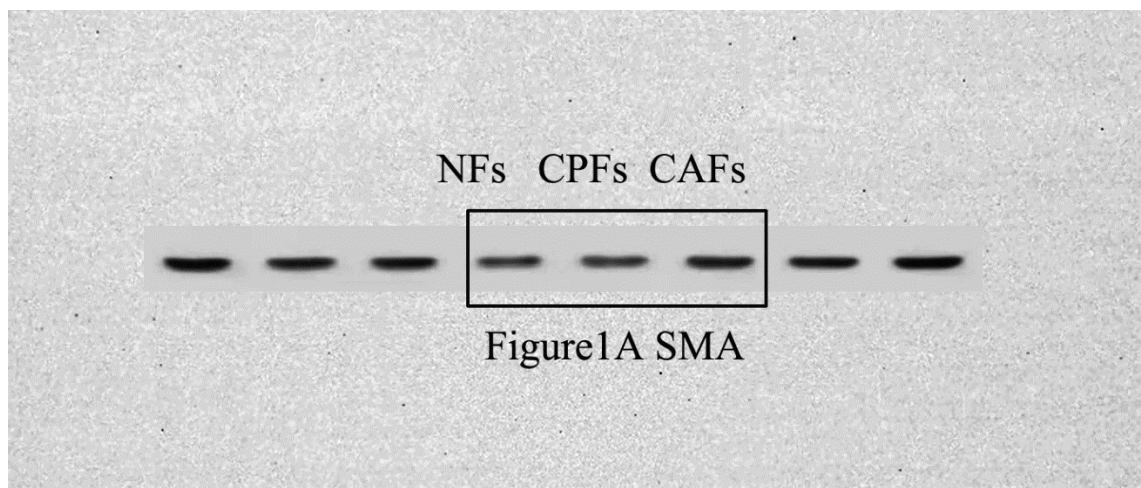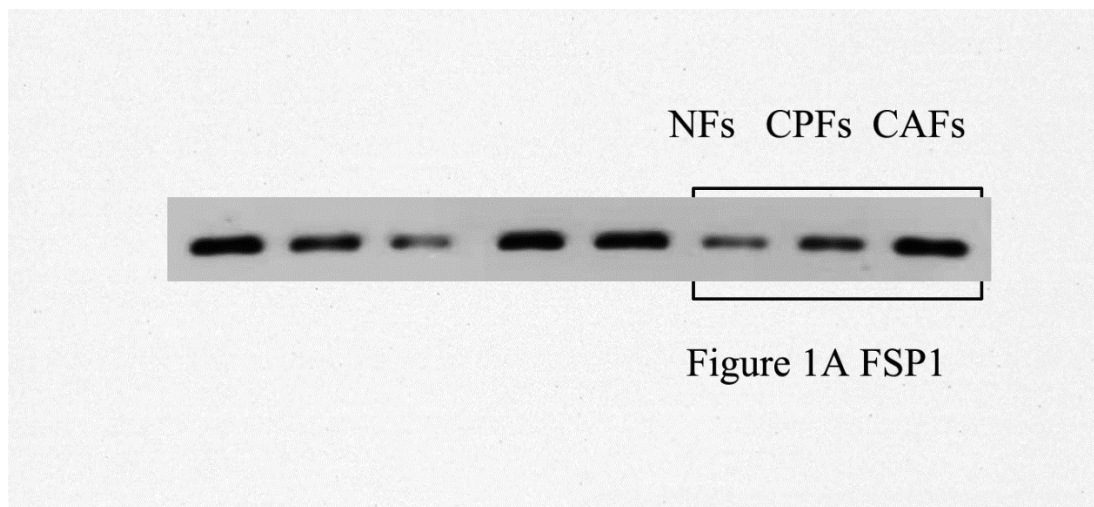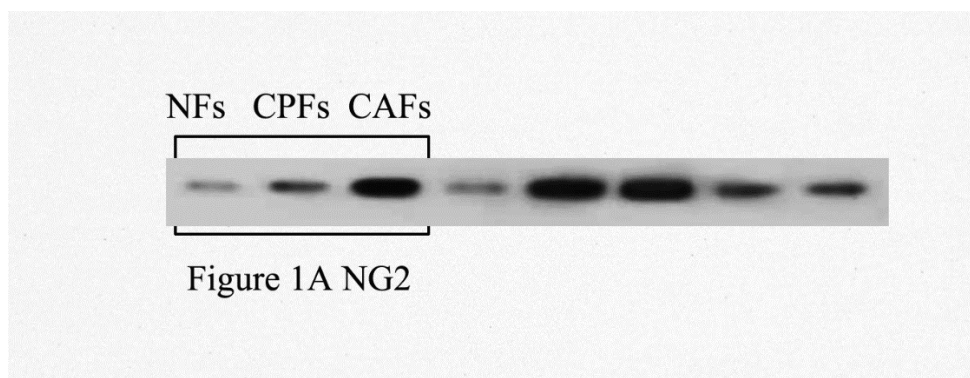

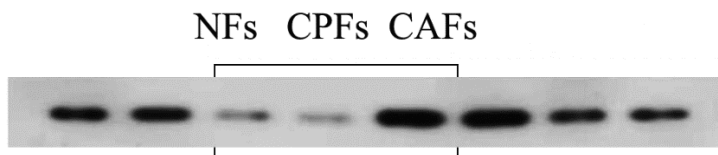

Figure 1A PDGF-β

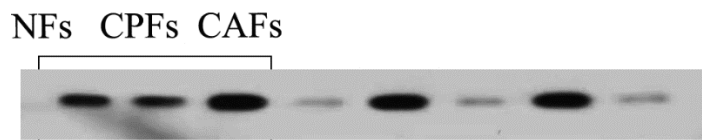

Figure 1J ROCK1

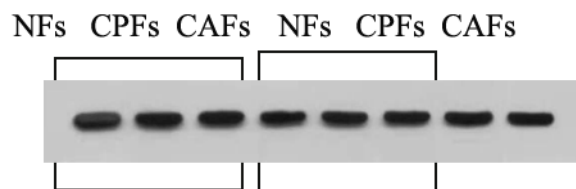

Figure 1A GAPDH Figure 1J GAPDH

Figure2

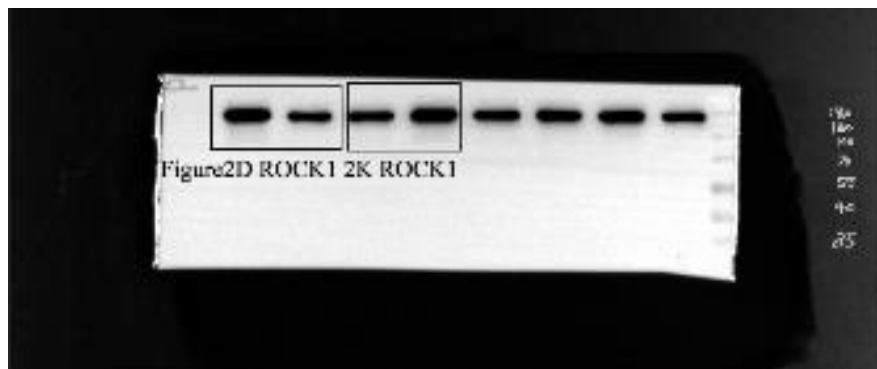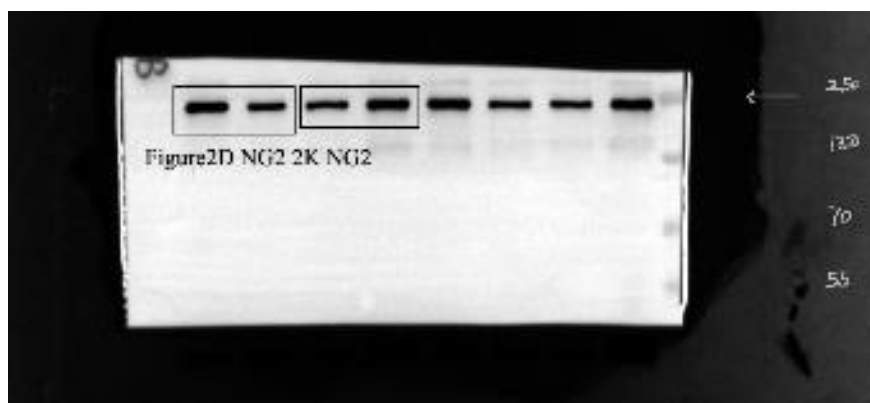

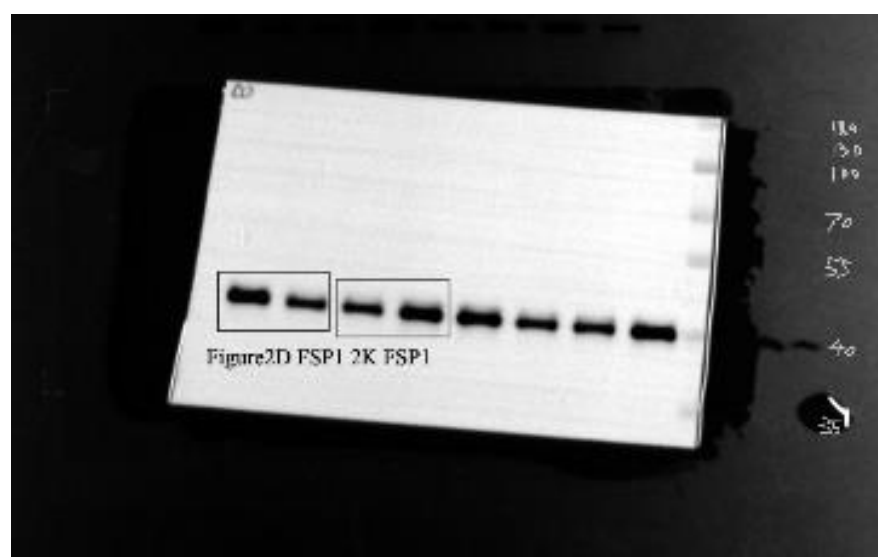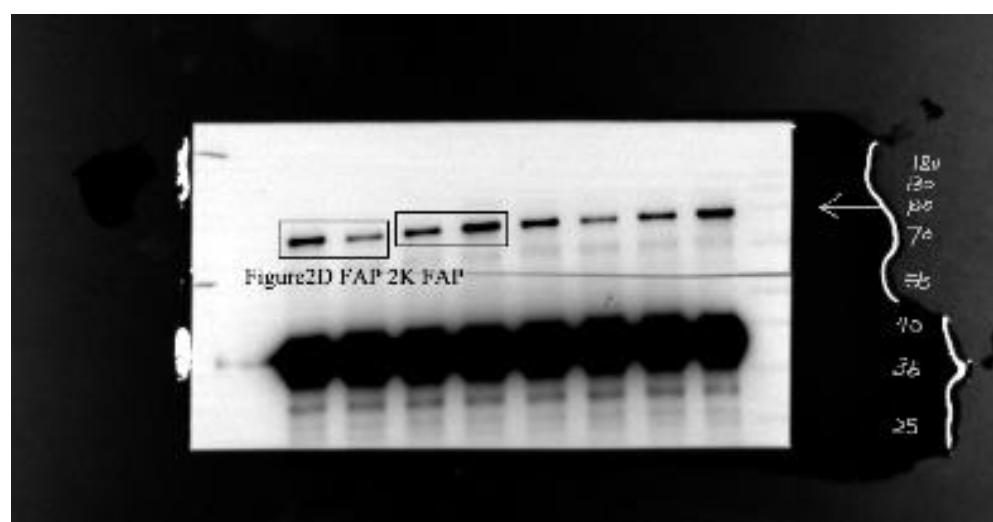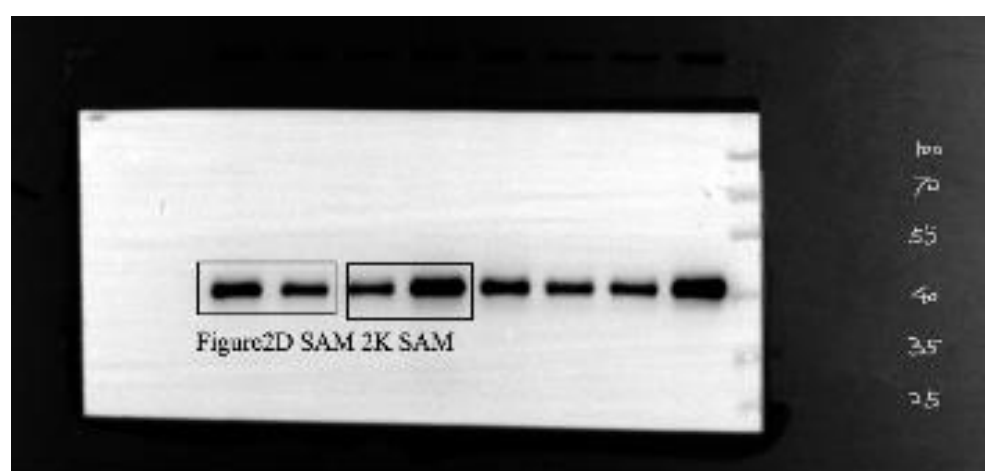

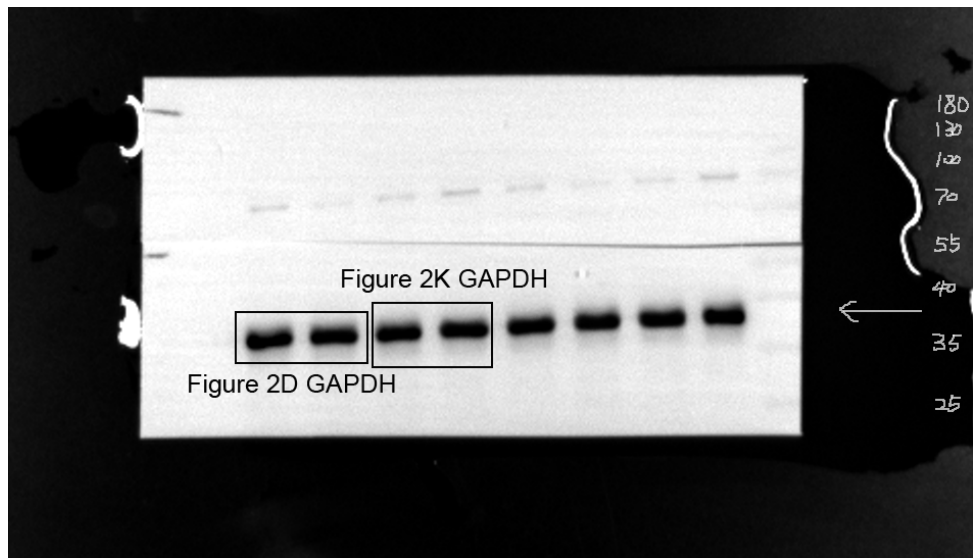

Figure3

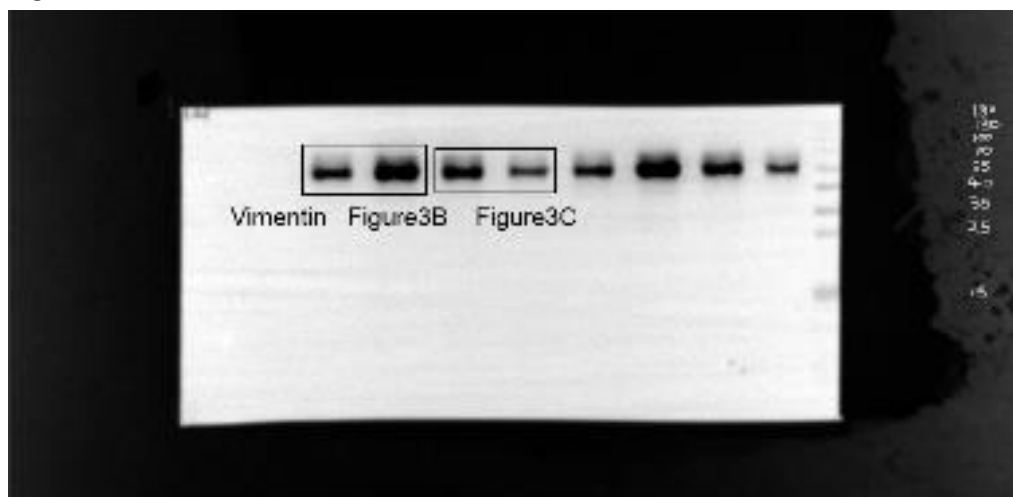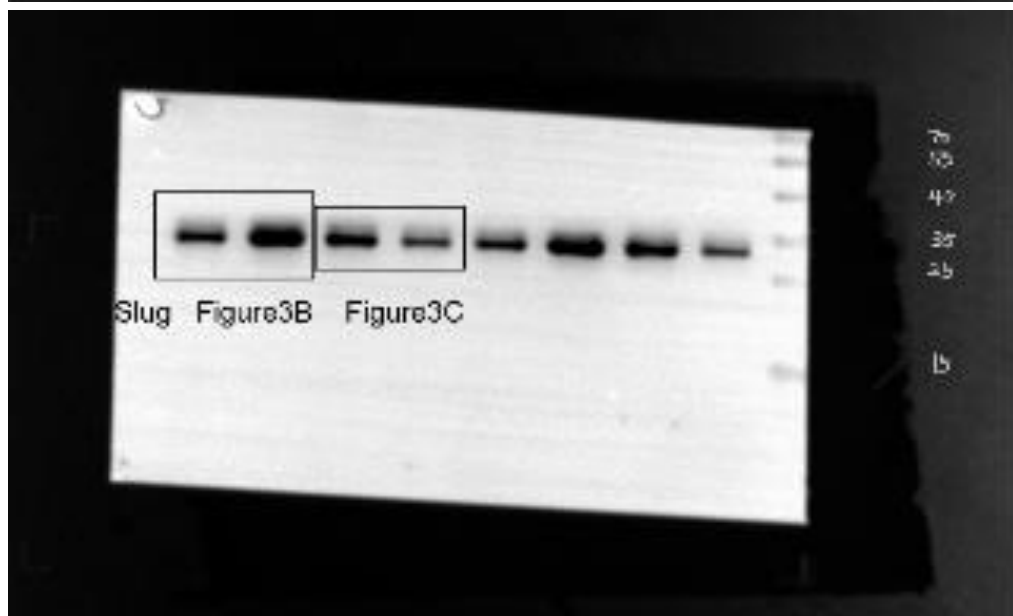

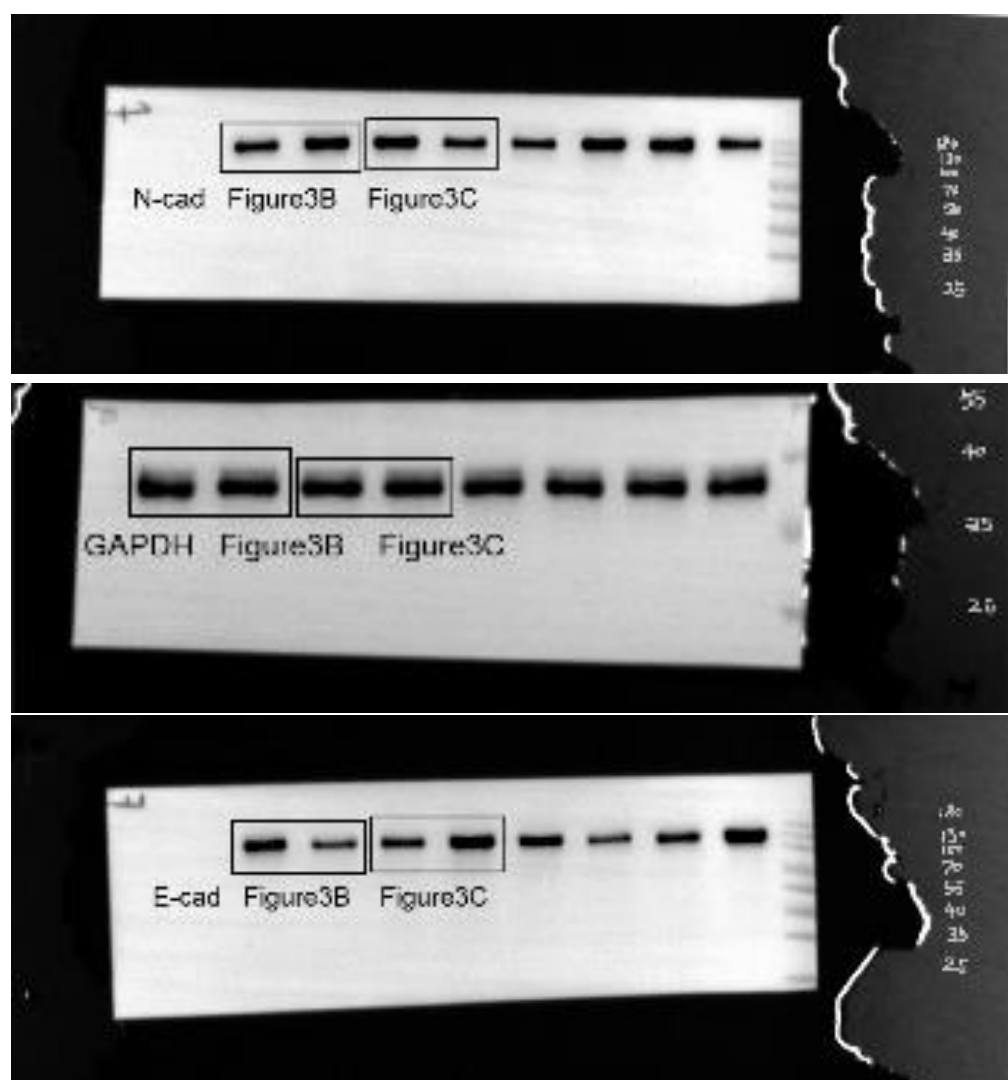

Figure 4

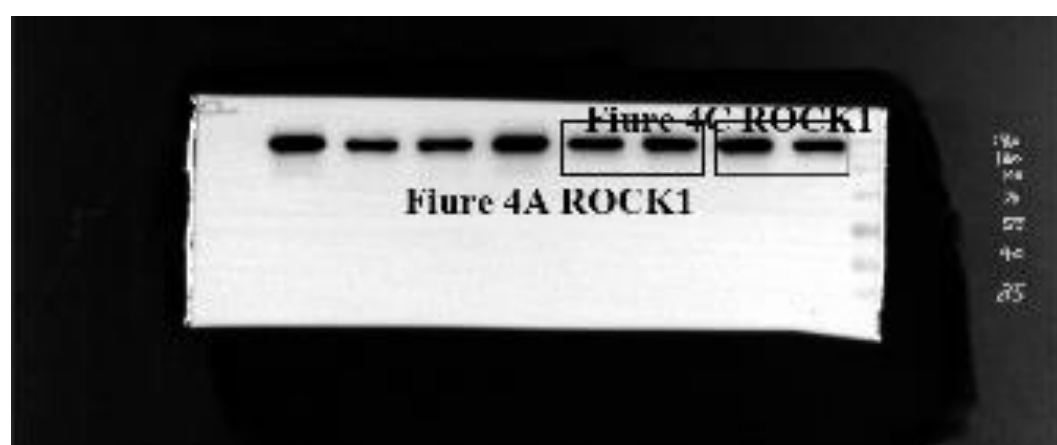

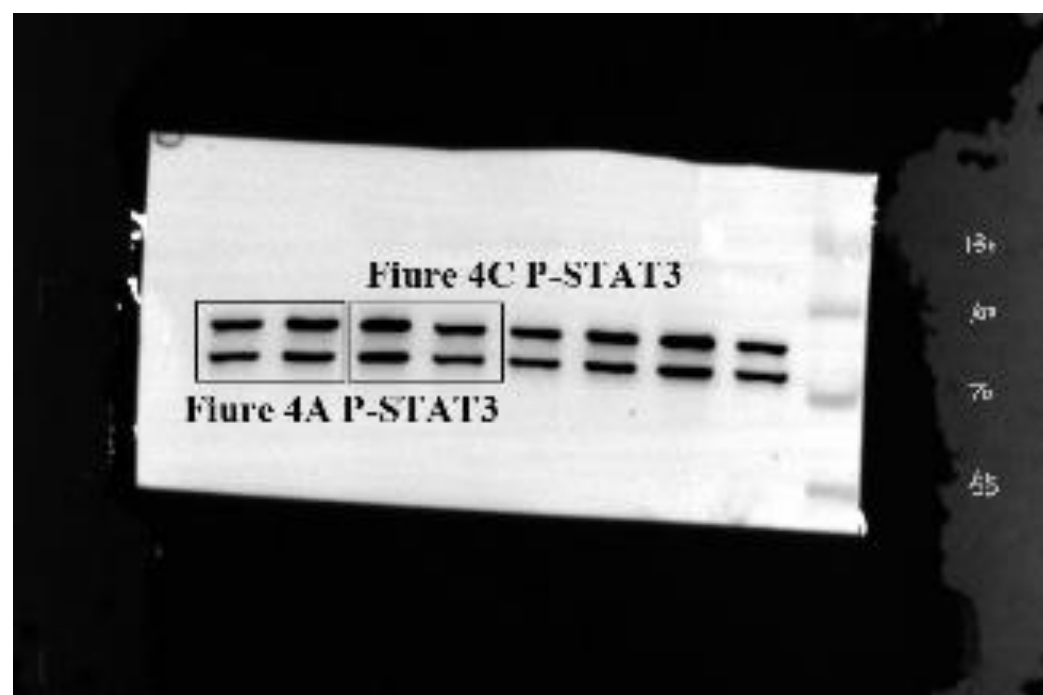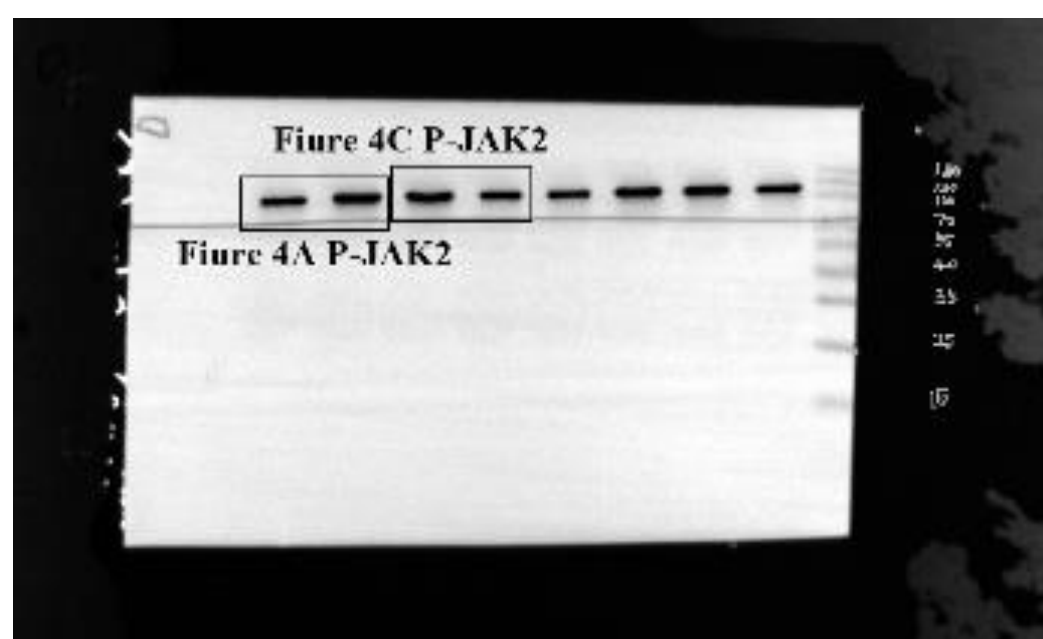

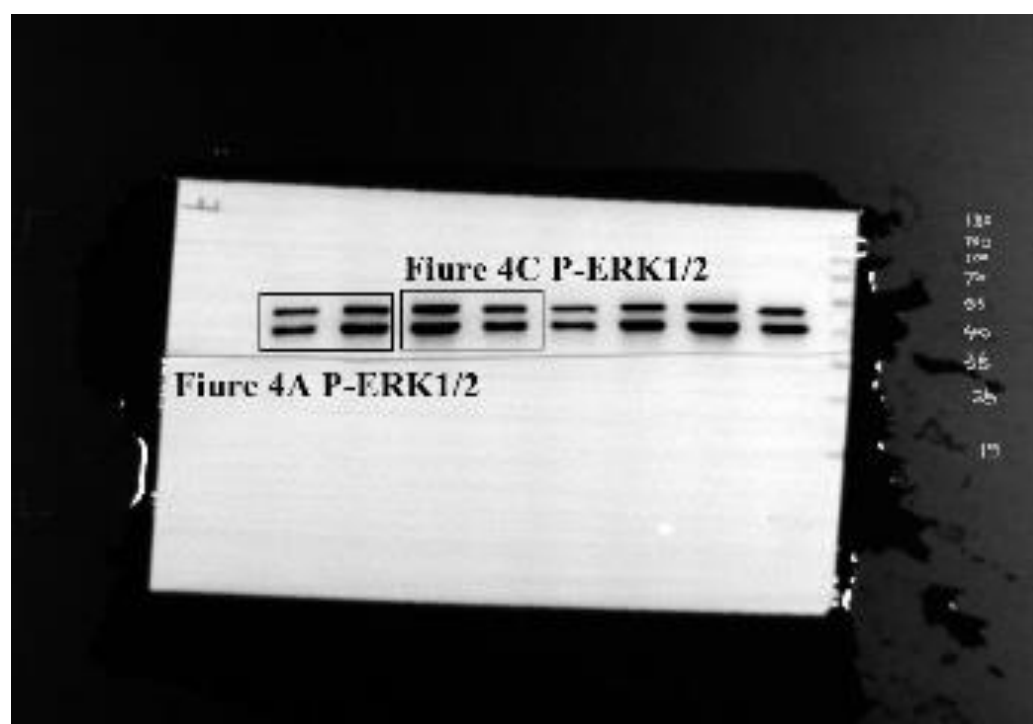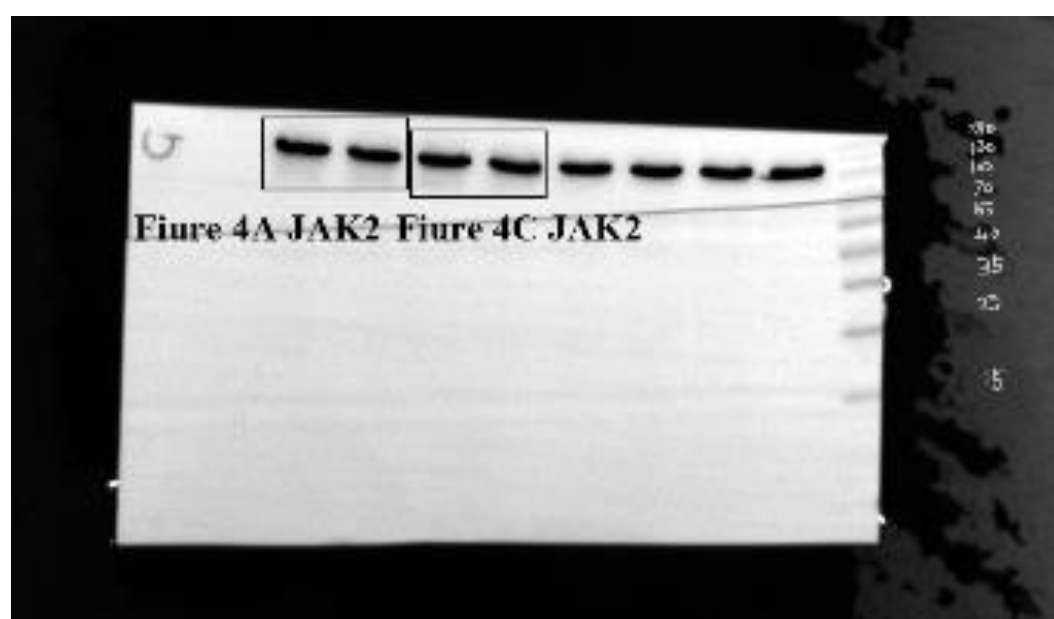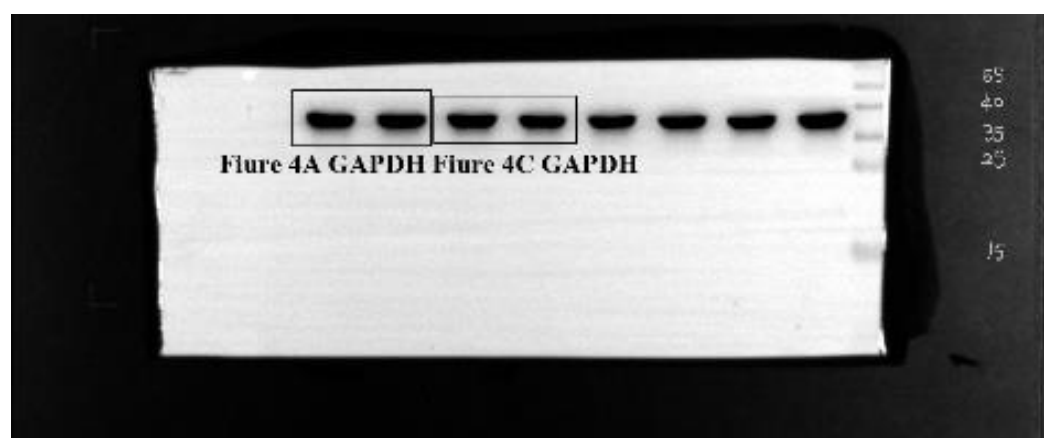

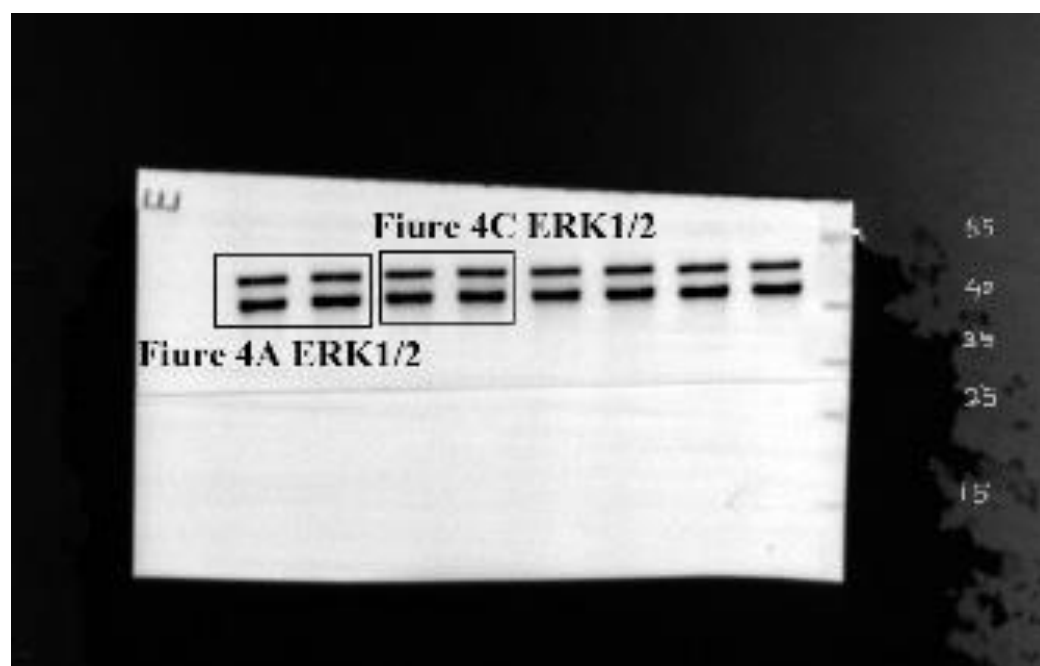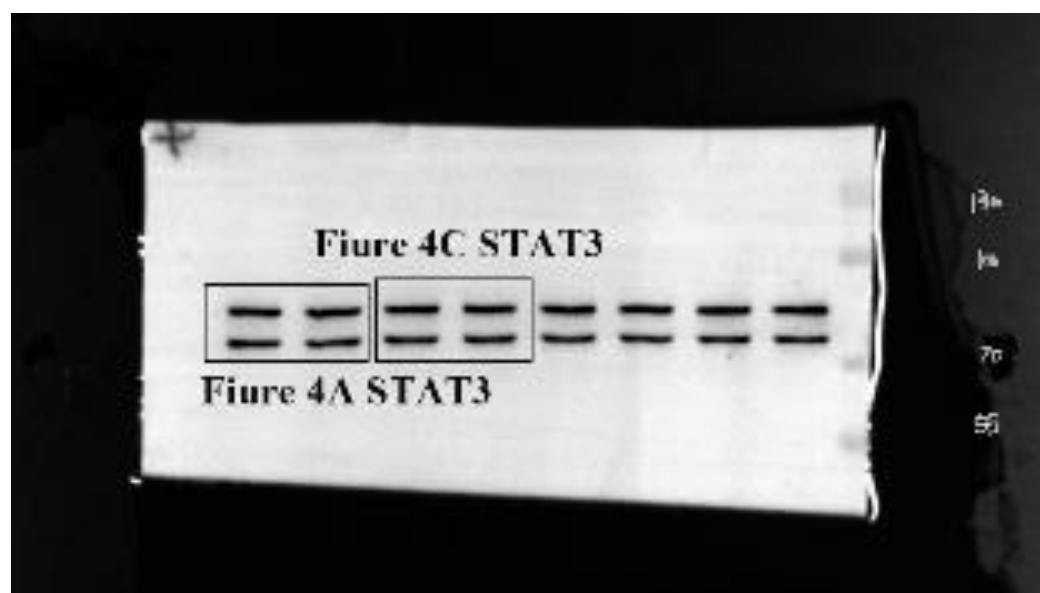

Figure 5-7

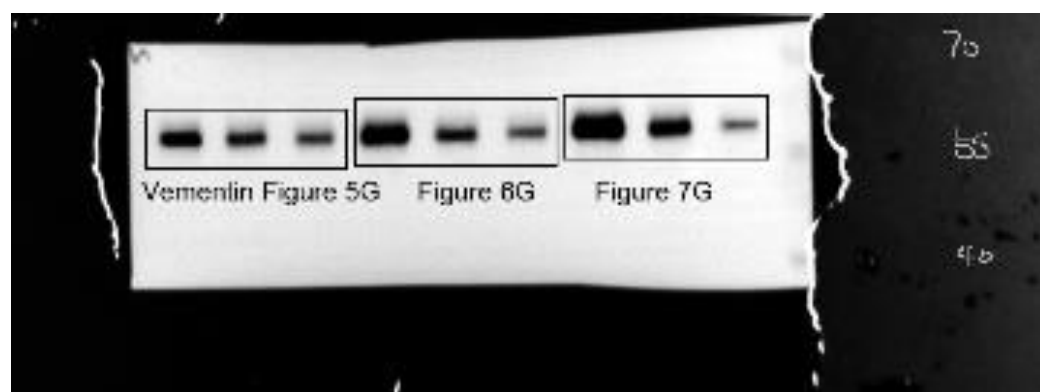

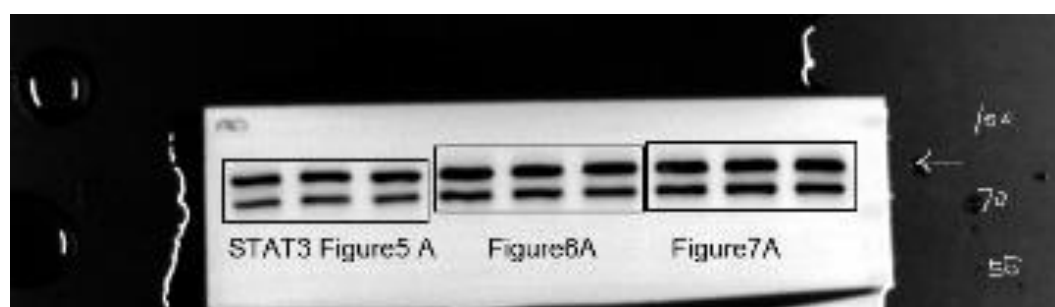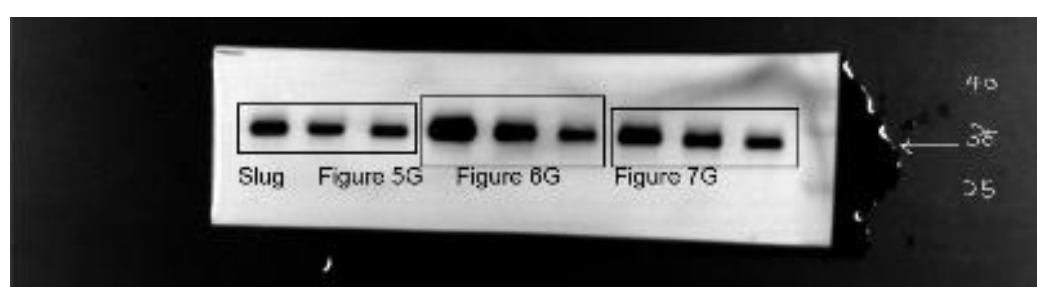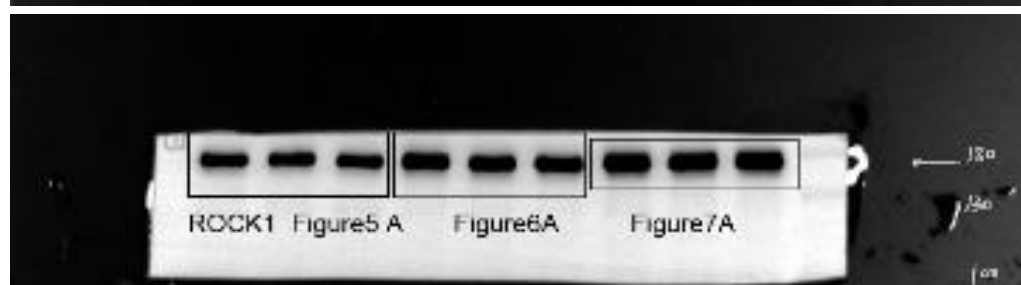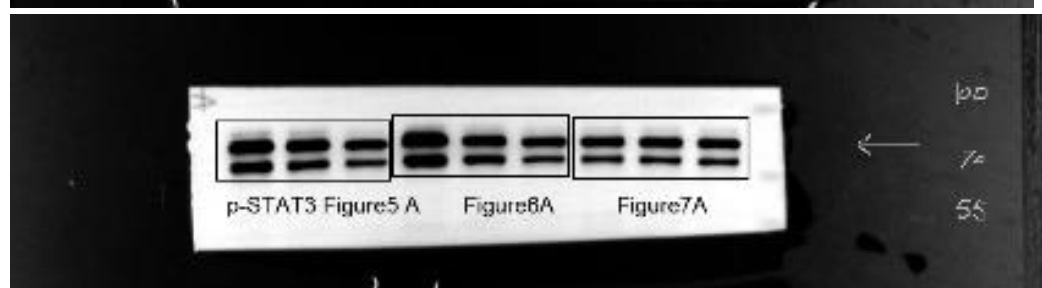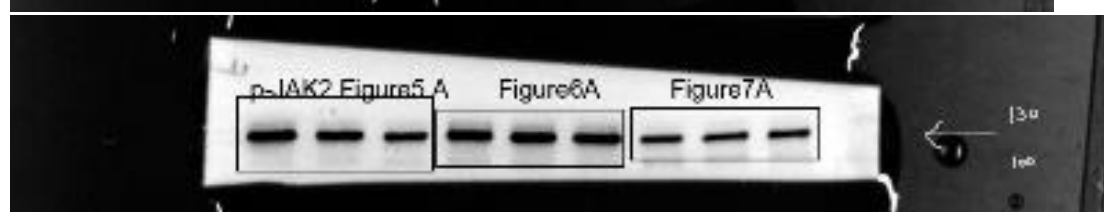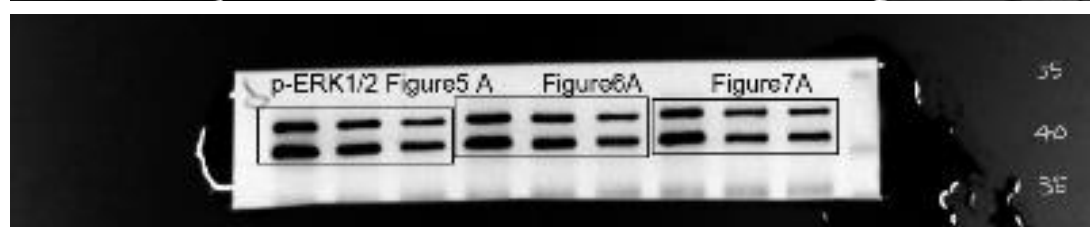

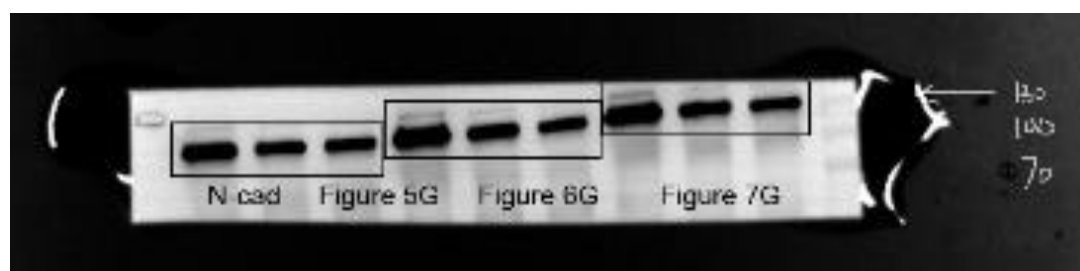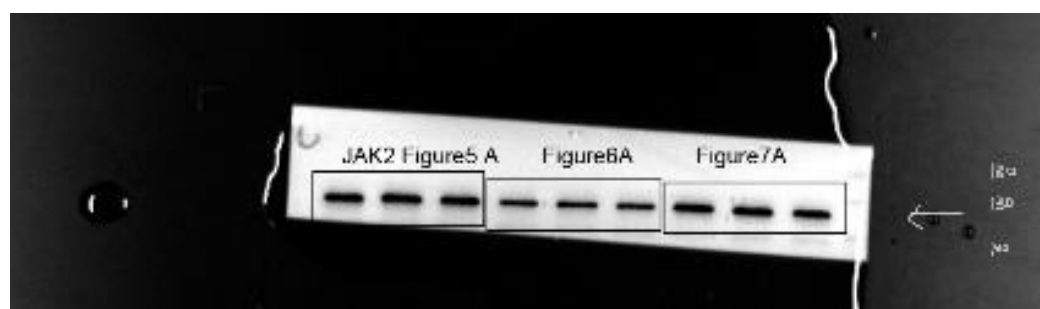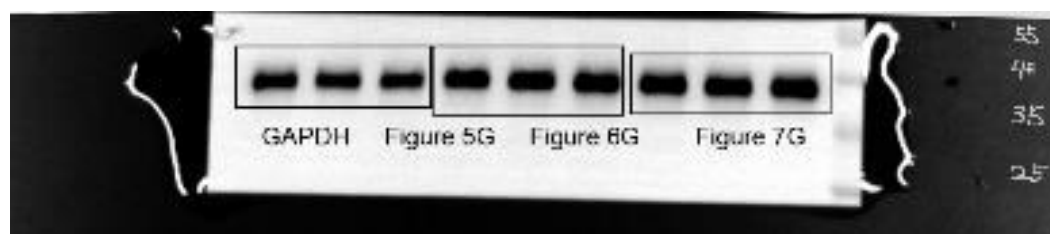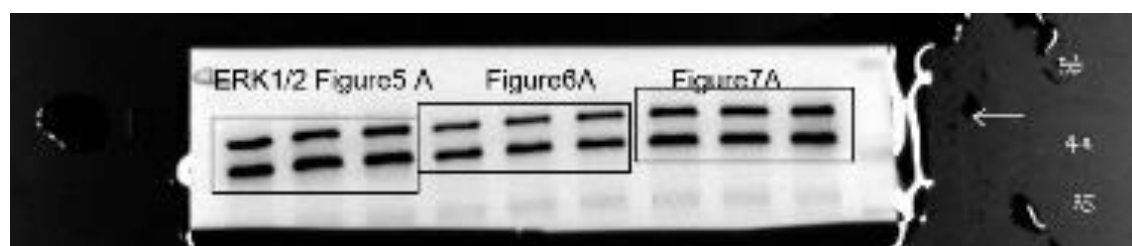

Supplement: Supplementary file 1 — Supplementary Material 1 [file 12935_2023_2911_MOESM1_ESM.pdf]
